# ER maleate is a novel anticancer agent in oral cancer: implications for cancer therapy

**DOI:** 10.18632/oncotarget.7751

**Published:** 2016-02-26

**Authors:** Guodong Fu, Raj Thani Somasundaram, Fatima Jessa, Gunjan Srivastava, Christina MacMillan, Ian Witterick, Paul G. Walfish, Ranju Ralhan

**Affiliations:** ^1^ Department of Medicine, Alex and Simona Shnaider Research Laboratory in Molecular Oncology, Endocrine Division, Mount Sinai Hospital, Toronto, Canada; ^2^ Department of Pathology and Laboratory Medicine, Mount Sinai Hospital, Toronto, Canada; ^3^ Department of Otolaryngology — Head and Neck Surgery, Joseph and Mildred Sonshine Family Centre for Head and Neck Diseases, Mount Sinai Hospital, Toronto, Canada; ^4^ Department of Medicine, Endocrine Division, Mount Sinai Hospital and University of Toronto, Toronto, Canada; ^5^ Department of Otolaryngology — Head and Neck Surgery, University of Toronto, Toronto, Canada

**Keywords:** anticancer agent, ER maleate, Syk / PLK1, OSCC, tumor xenografts

## Abstract

ER maleate [10-(3-Aminopropyl)-3, 4-dimethyl-9(10H)-acridinone maleate] identified in a kinome screen was investigated as a novel anticancer agent for oral squamous cell carcinoma (OSCC). Our aim was to demonstrate its anticancer effects, identify putative molecular targets and determine their clinical relevance and investigate its chemosensitization potential for platinum drugs to aid in OSCC management. Biologic effects of ER maleate were determined using oral cancer cell lines *in vitro* and oral tumor xenografts *in vivo*. mRNA profiling, real time PCR and western blot revealed ER maleate modulated the expression of polo-like kinase 1 (PLK1) and spleen tyrosine kinase (Syk). Their clinical significance was determined in oral SCC patients by immunohistochemistry and correlated with prognosis by Kaplan-Meier survival and multivariate Cox regression analyses. ER maleate induced cell apoptosis, inhibited proliferation, colony formation, migration and invasion in oral cancer cells. Imagestream analysis revealed cell cycle arrest in G_2_/M phase and increased polyploidy, unravelling deregulation of cell division and cell death. Mechanistically, ER maleate decreased expression of PLK1 and Syk, induced cleavage of PARP, caspase9 and caspase3, and increased chemosensitivity to carboplatin; significantly suppressed tumor growth and increased antitumor activity of carboplatin in tumor xenografts. ER maleate treated tumor xenografts showed reduced PLK1 and Syk expression. Clinical investigations revealed overexpression of PLK1 and Syk in oral SCC patients that correlated with disease prognosis. Our *in vitro* and *in vivo* findings provide a strong rationale for pre-clinical efficacy of ER maleate as a novel anticancer agent and chemosensitizer of platinum drugs for OSCC.

## INTRODUCTION

Oral squamous cell carcinoma (OSCC) is the major subset of head and neck cancer, which ranks as the sixth most common cancer worldwide [1]. Head and neck cancer patients diagnosed at stages I and II have five-year survival rates of 70% – 90% [2–4], while those diagnosed in stages III and IV have 50% survival, limited treatment options and poor prognosis [5]. Current treatments including primary surgery and/or a combination of chemo- and radio- therapy for OSCC patients are traumatic, disfiguring and drastically compromise their quality of life [6]. Chemotherapy (CT) using cisplatin/carboplatin, methotrexate or taxanes as single agents or in combination are given in recurrent or metastatic head and neck cancer; dose-limiting toxicities restrict their clinical utility [3, 7–10]. Monotargeted therapies including inhibitors of EGFR, STAT3, NFκB and mammalian target of rapamycin (mTOR) have shown limited efficacy [11–14]. There exists urgent need for development of new drugs for oral cancer. Small bioactive molecules are being explored as inhibitors of novel kinases as molecular therapeutic targets, including Spleen tyrosine kinase (Syk) and Polo-like kinase 1 (PLK1). Using quantitative high throughput assays, ER 27319 maleate [10-(3-Aminopropyl)-3,4-dimethyl-9(10H)-acridinone maleate] (ER maleate) was identified as one of the most effective cytotoxic agents for OSCC by screening six chemical libraries containing 5170 small molecule inhibitors [15].

Syk is a cytoplasmic tyrosine kinase with critical roles in B cell development, initiation of inflammatory responses, and acts as a pro-survival factor in cancers of both hematopoietic and epithelial origins [16, 17]. The expression of Syk is increased in head and neck cancer and associated with lymph node metastases and chemomigration of HNSCC cells [18]. PLK1 is an essential mitotic kinase that phosphorylates Ser/Thr residues in proteins and has pleiotropic roles in regulation of cell division, including effects on G_2_/M transition, centrosome maturation, mitotic spindle formation, chromosome segregation, and cytokinesis [19]. PLK1 is involved in checkpoint recovery and resuming cell cycle progression after DNA damage-induced cell cycle arrest [20]; it is overexpressed in human cancers [21, 22]. Clinical trials on small-molecule PLK inhibitors in solid tumors have been conducted [23–25] and BI6726 has provided a survival benefit for acute myeloid leukemia patients [26–28]. However, PLK1 as a critical mediator in oral cancer development remains to be examined.

In this study, ER maleate was identified as a novel anticancer agent in OSCC. ER maleate induced apoptosis, inhibited cell migration and invasion, cell proliferation by inducing cell cycle arrest in G_2_ phase and blocking cell division *in vitro*. Reduction of oral xenograft tumor volume with paralleling decrease in Syk and PLK1 expression, as well as chemosensitization of platinum drug *in vivo*, together with overexpression of Syk and PLK1 in clinical OSCCs provided evidence for development of ER maleate as a novel therapeutic agent for OSCC.

## RESULTS

### ER maleate is a small molecule inhibitor for OSCC

**Figure 1:**
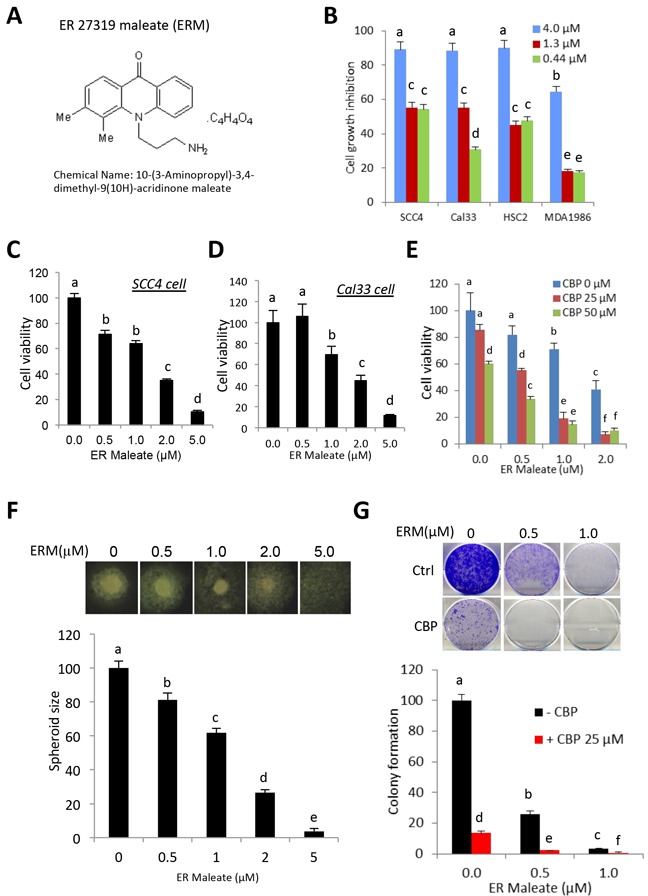
ER maleate inhibited cell proliferation, survival, spheroid formation and colony formation in OSCC cells **A.** The chemical structure of ER maleate. **B.** ER maleate showed cytotoxic effect using three doses (4μM, 1.33μM and 0.44μM) in SCC4, Cal33, HSC2 and MDA1986 cells from second round validation of 48 inhibitors (15). **C&D.** ER maleate inhibited cell proliferation in a dose dependent manner (0-5 μM) in SCC4 cells (C) and Cal33 cells (D) by MTT assay. **E.** ER maleate (0-2 μM) enhanced carboplatin (0-50 μM) inhibited cell proliferation in SCC4 cells by MTT assay. **F.** Spheroid formation. ER maleate incubation with SCC4 cells decreased the cell density and size of spheroids in a dose dependent manner (0-2 μM) and failed to form the spheroid at 5μM (lower panel). Representative SCC4 cell spheroid is shown from each group (upper panel). Data are represented as mean ±SEM relative to the control from three independent experiments. **G.** Colony formation assay. SCC4 cells were treated with ER maleate (0.5 – 1 μM), carboplatin (CBP, 25μM) or in combination of ER maleate and carboplatin for 9 days. Colonies formed were stained and counted. Histogram analysis showed a significant reduction in colony forming ability in ER maleate treated cells with a further reduction in combination of ER maleate and carboplatin treatment (lower panel). Representative stained colonies were shown from each group (upper panel). Data are represented as mean ±SEM relative to the control from three independent experiments. Treatment groups denoted by different letters represent a significant difference at *p*<0.05 (ANOVA followed by Fisher's LSD test).

ER maleate (Figure 1A) emerged as a promising inhibitor of OSCC through the kinase inhibitor library screen [15]. Further verification of ER maleate using cytotoxicity assays with three-fold dilutions (4, 1.3 and 0.44 μM) in four OSCC cell lines (SCC4, Cal33, MDA1986 and HSC2) confirmed its cytotoxic potential (Figure 1B).

### ER maleate inhibited cell proliferation, survival, spheroid formation and colony formation

To determine the anti-proliferative effects of ER maleate in OSCC, cell based assays were performed by incubating SCC4 and Cal33 cells with ER maleate (0-5 μM). ER maleate treatment resulted in a dose dependent decrease in cell viability with 64% cell viability at 1μM and 35% at 2μM in SCC4, and 69% cell viability at 1μM and 45% at 2μM in Cal33 cells (Figure 1C, 1D). The 50% inhibitory concentration (IC_50_) of ER maleate determined using the line of best fit was 2.1μM for SCC4 and 2.6μM for Cal33 cells. Carboplatin, a commonly used anticancer drug, showed inhibition of SCC4 cell viability (Figure 1E). Oral cancer cells spheroid formation, an *in vitro* 3D culture model, showed incubation of SCC4 cells with ER maleate decreased the cell density and size of spheroids in a dose dependent manner (0-2 μM) (Figure 1F). ER maleate (0.5-1 μM) treatment of OSCC cells for 48 h significantly inhibited their colony formation potential in long term cultures (9 days) (Figure 1G).

### ER maleate inhibited cell invasion and migration potential in OSCC cells

**Figure 2:**
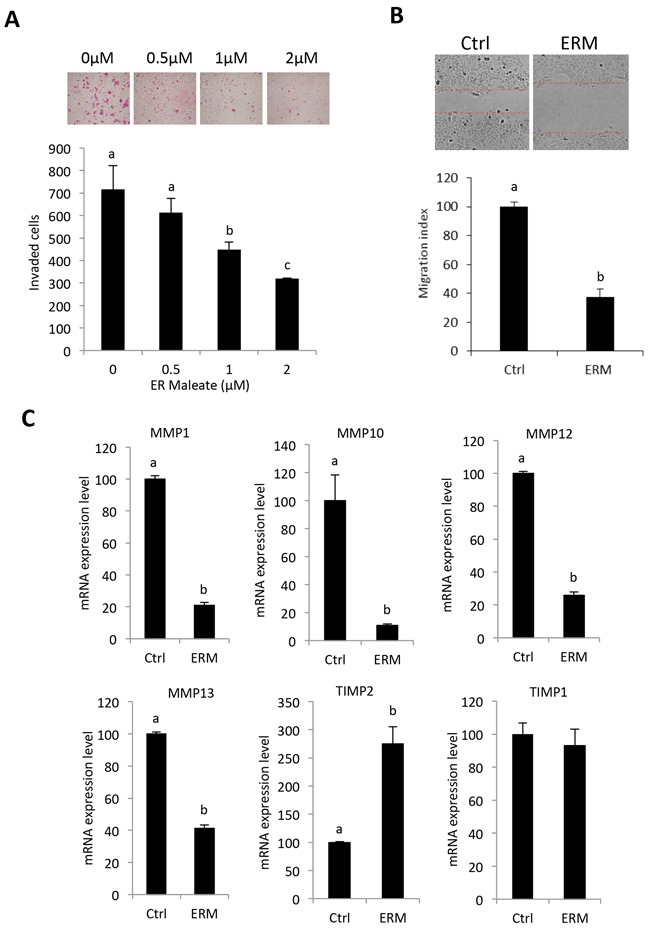
ER maleate inhibited cell invasion and migration potential, and modulated the expression of TIMP-MMPs in OSCC cells **A.** ER maleate significantly inhibited invasive capability of SCC4 cells in a dose dependent manner (0 – 2 μM) after 24 h incubation by transwell invasion assay. Bar graphs show the decrease in invaded cell number with ER maleate treatment in a dose dependent manner. **B.** ER maleate significantly suppressed cell migration to the wound area in SCC4 cells in comparison with vehicle control cells in 24 h by wound healing assays. Histogram analysis showing significantly low number of cells in wound of ER maleate treated cells. **C.** ER maleate treatment decreased the expression of MMP-1, MMP-10, MMP-12 and MMP-13, while TIMP-2 expression increased with no significant change in TIMP-1 at the mRNA level in SCC4 cells analyzed by illumine mRNA profiles. The bar graph data presented as mean ± SEM; groups denoted by different letters represent a significant difference at *p* < 0.05(ANOVA followed by Fisher's LSD test).

Transwell matrigel invasion assay showed ER maleate significantly inhibited invasive capability of SCC4 cells in a dose dependent manner (0 – 2 μM) within 24 h (Figure 2A). Similarly, wound healing assay revealed ER maleate significantly suppressed cell migration to the wound area in SCC4 cells in 24 h (Figure 2B). Matrix metalloproteinases (MMP) MMP1, MMP10, MMP12 and MMP13 expression were decreased at mRNA level, while tissue inhibitor of metalloproteinase2 (TIMP2) expression increased with no significant change in TIMP1 (Figure 2C).

### ER maleate induced cell apoptosis

**Figure 3:**
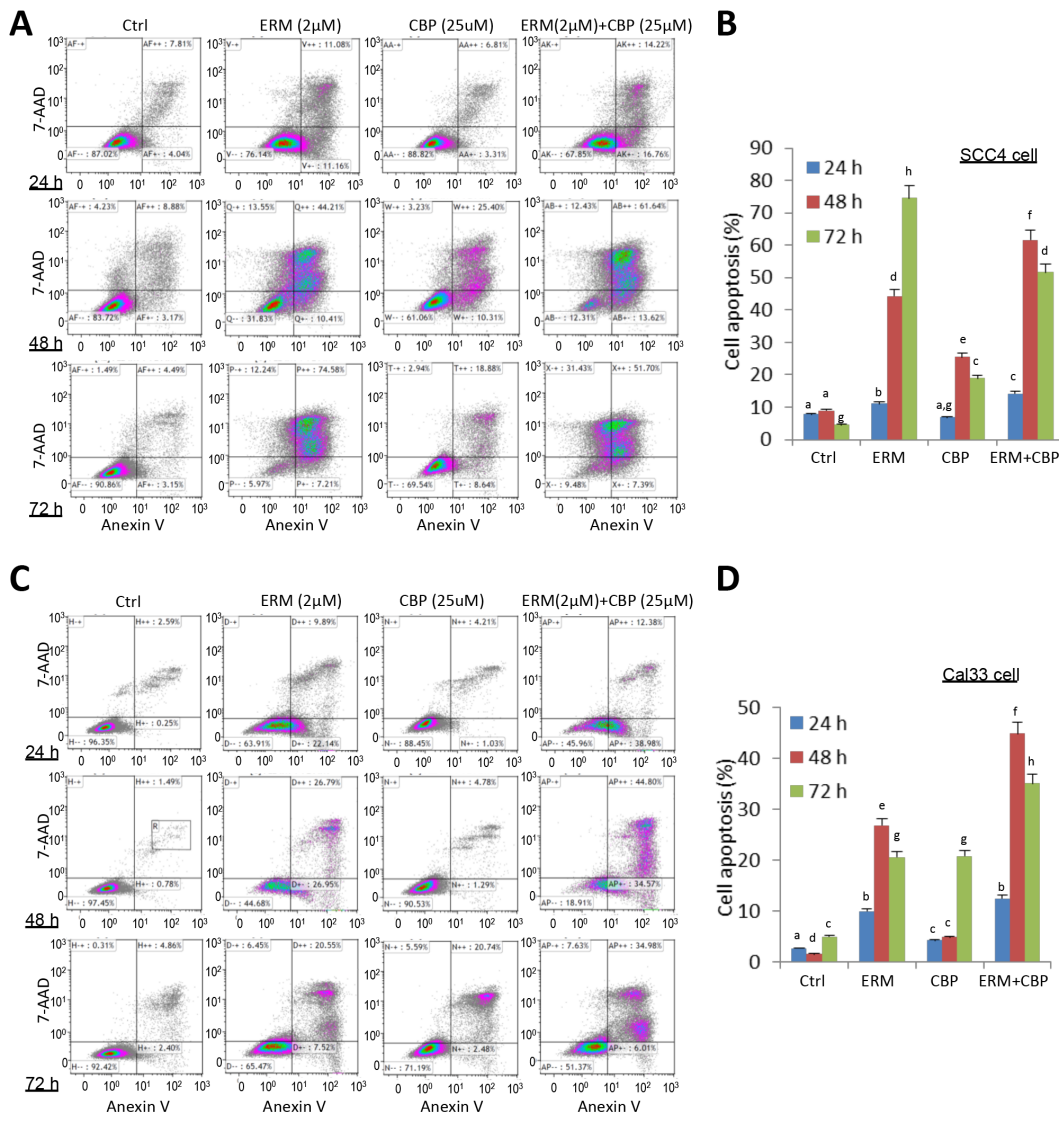
ER maleate induced apoptosis in OSCC cells by Annexin-V and 7-ADD double staining assay **A.** A significant increase in cell apoptosis/death was observed in SCC4 cells on treatment with ER maleate (2μM), or CBP (25μM) alone, or their combination for 24h, 48h and 72h, respectively. CBP treatment induced apoptotic cell population and this induction was further enhanced by combining with ER maleate. **B.** Histogram showed the change in apoptotic cell percentage of SCC4 cells on treatment with ER maleate (2μM), or CBP (25μM) alone or their combination. **C.** An increase in apoptosis was also observed in Cal33 cells on treatment with ER maleate, or CBP (25μM) alone or their combination for 24h, 48h and 72h, respectively. CBP treatment induced apoptotic cell population and this induction was further enhanced by combining with ER maleate. **D.** Histogram showed the change in apoptotic cell percentage of Cal33 cells on treatment with ER maleate (2μM), or CBP (25μM) alone or their combination. The bar graph data were presented as mean ± SEM; groups denoted by different letters represent a significant difference at *p* < 0.05 (ANOVA followed by Fisher's LSD test).

**Figure 4:**
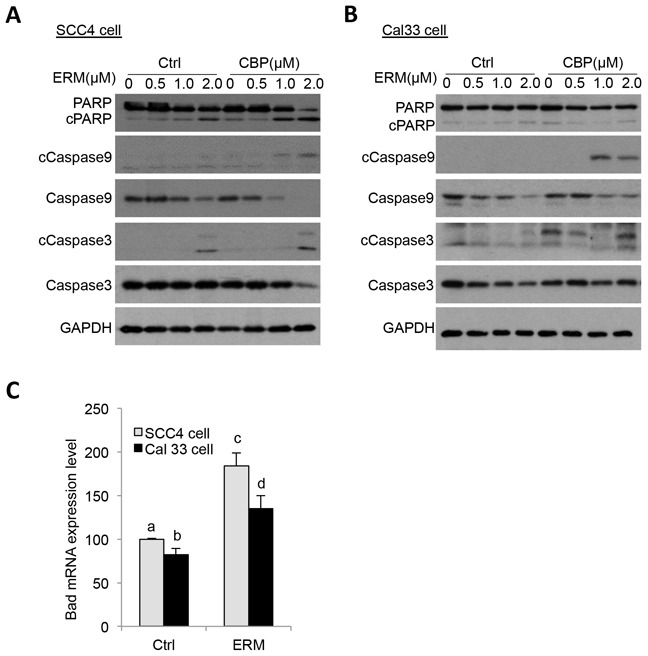
ER maleate induced cleavage of PARP, caspase9 and caspase3 in OSCC cells **A, B.** ER maleate treatment for 24 h induced the expression of cleaved PARP, and caspase3 and reduced levels of full length caspase 9 and caspase 3 in SCC4 (A) and Cal33 cells (B) by western blot analysis. **C.** ER maleate induced Bad mRNA expression in both SCC4 and Cal33 cells at 24 h. The bar graph data were presented as mean ± SEM; groups denoted by different letters represent a significant difference at *p* < 0.05 (ANOVA followed by Fisher's LSD test).

ER maleate (2μM) showed a significant increase in apoptosis in SCC4 and Cal33 cells by Annexin-V and 7-ADD double staining assay (Figure 3A–3D). ER maleate treatment resulted in increased cell apoptosis, 11.08%, 44.21% and 74.58% in SCC4 cells at 24 h, 48 h and 72 h, respectively (Figure 3A, 3B). Similar increase in apoptosis was also observed in Cal33 cells with ER maleate treatment (Figure 3C, 3D). ER maleate also induced cleavage of PARP and increased the level of cleaved PARP. Similarly, the levels of full length caspase9 and caspase3 were decreased by ER maleate treatment in a dose dependent manner (0-2 μM) (Figure 4A, 4B), and the induction of cleaved caspase3 was detectable in SCC4 cells, while the cleaved caspase9 could not be visualized (Figure 4A, 4B), confirming ER maleate induced apoptosis through PARP, caspase3 and caspase9 pathway. Their expression changes were quantitated and shown as histograms (Supplementary Figure S1A–S1L). The pro-apoptotic *BAD* expression was induced at mRNA level in both SCC4 and Cal33 cells treated with ER maleate for 24 h (Figure 4C).

### ER maleate blocked cell division and induced polyploidy

**Figure 5:**
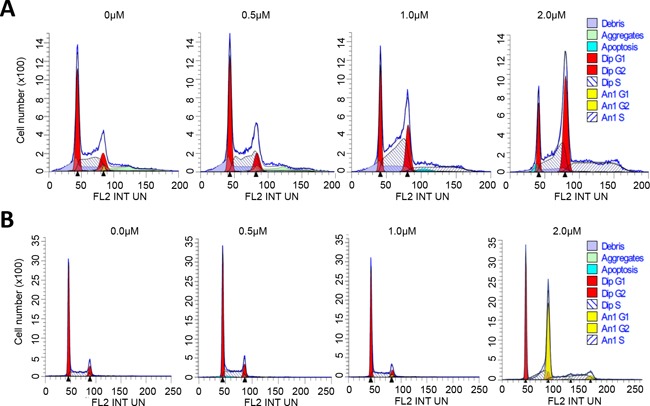
ER maleate arrested cell in G2 phase and induced polyploid population **A.** FACS analysis of SCC4 cells. ER maleate treatment decreased the diploid fraction from 95.42% to 70.82%, whereas it increased polyploid population from 4.58% to 29.18% in a dose dependent manner (0 – 2 μM, Supplementary Table S1). For the diploid cells, cell population in G_2_ phase was increased from 15.37% to 43.44% and in G_1_ phase decreased from 46.11% to 16.56% in a dose dependent manner (0 – 2 μM) with ER maleate treatment of SCC4 cells for 48 h through Modfit analysis. For the polyploid cell population, most cells (99.68%) accumulate in S phase on treatment with ER maleate at 2μM. **B.** FACS analysis of Cal33 cells. ER maleate decreased the diploid population from 100% to 43.86% but increased the polyploid population from 0 to 56.14% in Cal33 cells with ER maleate treatment (0-2 μM) for 48 h (Supplementary Table S2).

**Figure 6:**
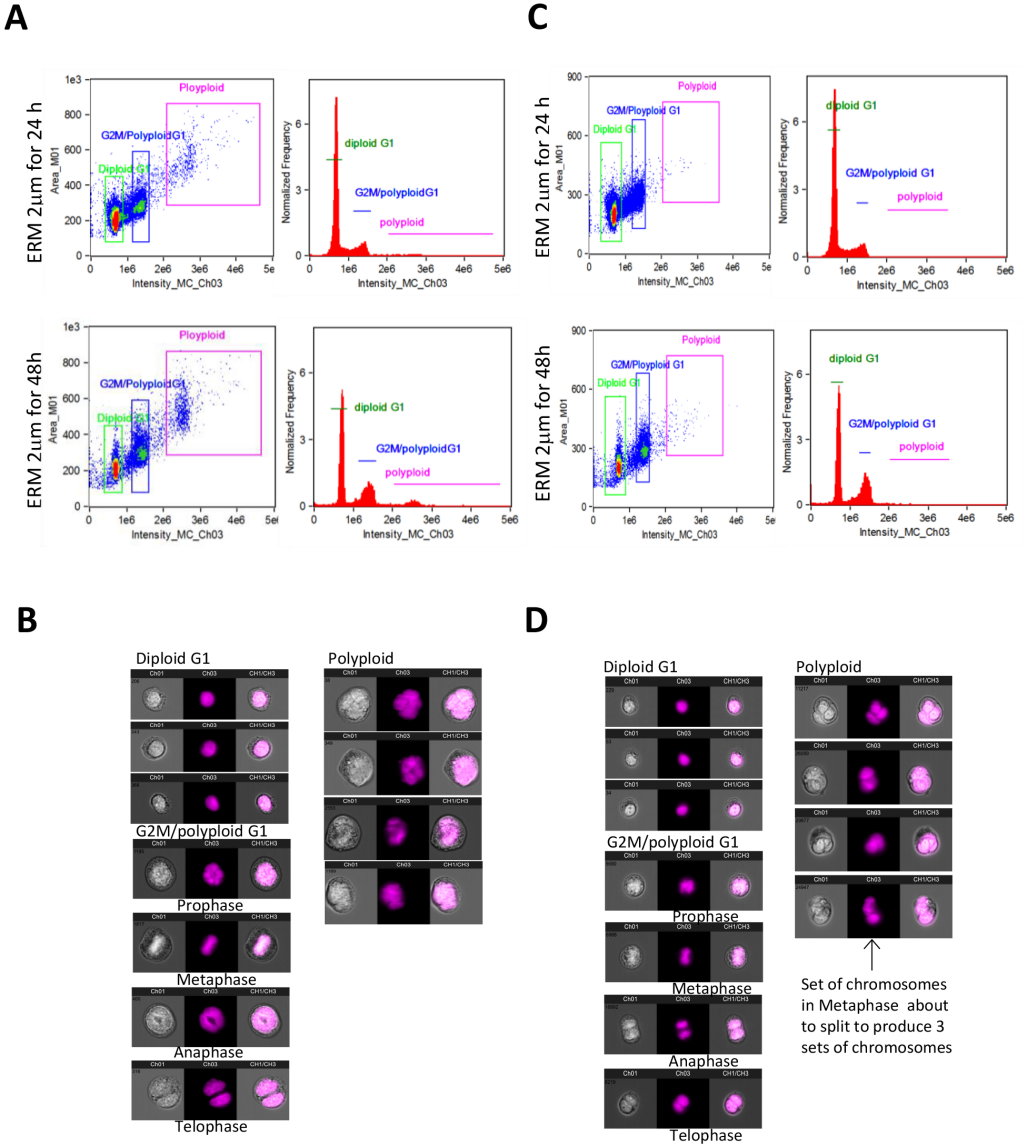
ImageStream FACS of OSCC cells **A.** Imagestream and Ideas program analysis showed ER maleate induced the tetraploid/anueploid (polyploid) cell population in SCC4 cell. Two dimensional plot shows the cell size and DNA content (*right panel*) and histogram shows cell DNA content (*left panel*) in SCC4 cells at 24 h (*Upper panel*) and 48 h (*Bottom panel*); **B.** Imagestream nuclear morphology of SCC4. Cell nuclei stained with PI and run on Amnis Imagestream MKII reveal a significant increase in tetraploid/anueploid (polyploid) cell population in SCC4 cells; **C.** Imagestream and Ideas program analysis showed ER maleate induced the tetraploid/anueploid (polyploid) cell population in Cal33 cell. Two dimensional plot shows the cell size and DNA content (*Right panel*) and histogram shows cell DNA content (*Left panel*) in Cal33 cells at 24 h (*Upper panel*) and 48 h (*Bottom panel*). **D.** Imagestream nuclear morphology of Cal33. Cell nuclei stained with PI and run on Amnis Imagestream MKII reveal a significant increase in tetraploid/anueploid (polyploid) cell population in Cal33 cells.

To further characterize ER maleate induced anti-proliferative effects on cell cycle, flow cytometry (FACS) using propidium iodide (PI) staining was performed. Modfit analysis showed ER maleate decreased diploid cell fraction and increased polyploid population in a dose dependent manner (Figure 5A, Supplementary Table S1). For diploid cells, cell population was increased in G_2_ phase from 15.37% to 43.44% and decreased in G_1_ phase from 46.11% to 16.56% in SCC4 cells treated with ER maleate in a dose dependent manner (0 – 2 μM) for 48 h (Figure 5A, Supplementary Table S1). For polyploid cell population, most cells (99.68%) accumulated in S phase but did not continue cell cycling on ER maleate (2μM) treatment for 48 h (Supplementary Table S1). Similarly, ER maleate decreased diploid fraction and increased polyploid population in Cal33 cells (Figure 5B, Supplementary Table S2). In both diploid and polyploid Cal33 cells, S phase fraction was also increased (Figure 5B, Supplementary Table S2). Imagestream analysis showed increases in cell size, DNA content, and number of polyploid cells with multiple nuclei, including tetraploid and anueploid cells in both SCC4 and Cal33 cells (Figure 6A–6D), providing image based evidence that DNA synthesis and replication in oral cancer cells continued, but cell division was inhibited and eventually resulted in cell death. These observations consistently support ER maleate inhibited cell proliferation (Figure 1C, 1D) and induced apoptosis in SCC4 and Cal33 cells (Figure 3 & 4).

### ER maleate inhibited gene expression of *PLK1* and *SYK* at mRNA level

**Figure 7:**
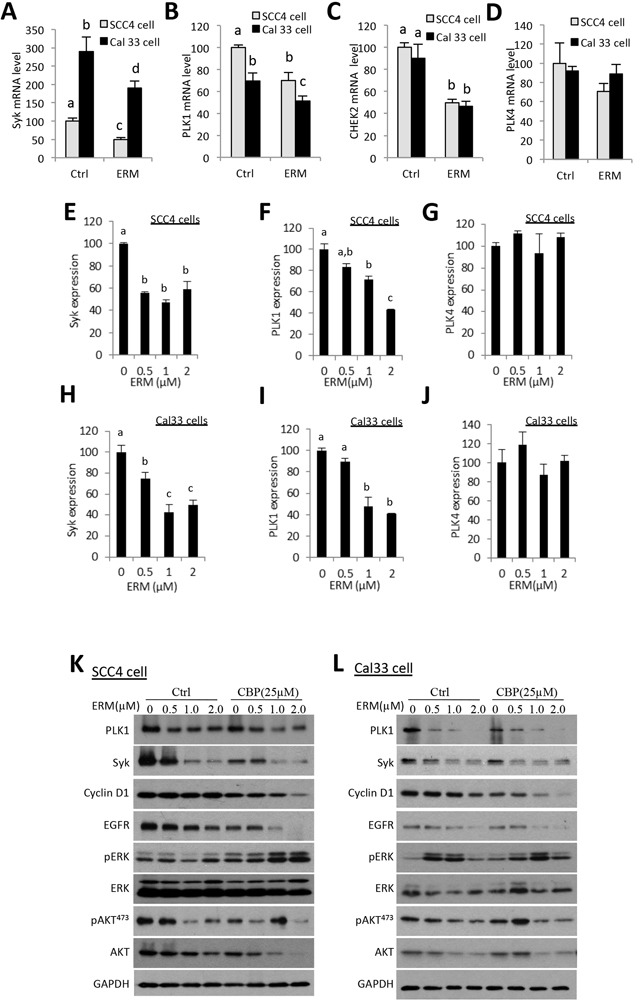
ER maleate inhibits the expression of Syk, PLK1, and CHEK2 and modulates PI3K/Akt signaling in OSCC cells **A–D.** Illumin mRNA profiling revealed ER maleate down-regulated gene expression of Syk (A), PLK1 (B), and CHEK2 (C) at mRNA level, but not PLK4 expression (D) in both SCC4 and Cal33 cells. **E–J.** Real-time PCR quantification showed the expression of Syk and PLK1was decreased in a dose dependent manner in SCC4 (E, F) and Cal33 cells (H, I) without significant change in PLK4 (G, J) in both SCC4 and Cal33 cells. The bar graph data were presented as mean ± SEM; groups denoted by different letters represent a significant difference at *p* < 0.05 (ANOVA followed by Fisher's LSD test). **K, L.** The expression of PLK1, Syk and Cyclin D1 was decreased in a dose dependent manner in SCC4 (K) and Cal33 cells (L) after treatment with ER maleate (0-2 μM) with or without CBP (25μM) for 48 h. Both phosphorylation level of Akt and the expression of total Akt were suppressed with ER maleate treatment in SCC4 (K) and Cal33 cells (L). EGFR kinase was also decreased at protein level by ER maleate in SCC4 (K) and Cal33 cells (L). GAPDH served as a loading control.

To unravel potential molecular targets, Illumina mRNA profiling showed that ER maleate inhibited gene expression of *SYK* (Figure 7A), *PLK1* (Figure 7B), and tumor suppressor gene *CHEK2* (Figure 7C) at mRNA level, but not *PLK4* expression (Figure 7D). Consistently, real time qPCR assay showed *PLK1* and *SYK* gene expression were suppressed by ER maleate in a dose dependent manner in both cells without significant change in *PLK4* expression (Figure 7E–7J).

### ER maleate inhibited PLK1, Syk, and PI3K/Akt signaling

Consistent with the change at mRNA level, the expression of PLK1 and Syk proteins was decreased by ER maleate (0-2 μM) in both SCC4 and Cal33 cells (Figure 7K, 7L), and also quantitated and shown as histograms (Supplementary Figure S2A–S2D). ER maleate also suppressed the cell proliferation marker cyclin D1 expression (Figure 7K–7L and Supplementary Figure S2E, S2F). To further reveal signaling pathways involved in ER maleate action, PI3K/Akt, mTOR, ERK and EGFR signaling pathways were examined in both cell lines. Both the phosphorylation level of Akt (pAkt^473^) and expression of total Akt were suppressed, whereas pERK was induced, by ER maleate treatment for 24 h. EGFR, an important mediator of EGFR signaling in oral cancer development, was also decreased by ER maleate (Figure 7K, 7L, and Supplementary Figure S2G, S2H). ER maleate treatment reduced the level of pAkt^473^ and pAkt^308^ in a time dependent manner in SCC4 and Cal33 cells, respectively (Supplementary Figure S3A, S3B). Phosphorylated mTOR (pmTOR), pS6 and ERK (pERK) were transiently induced then decreased by ER maleate in both cells (Supplementary Figure S3A, S3B).

### PLK1 and Syk partially rescued ER maleate-reduced cell viability

**Figure 8:**
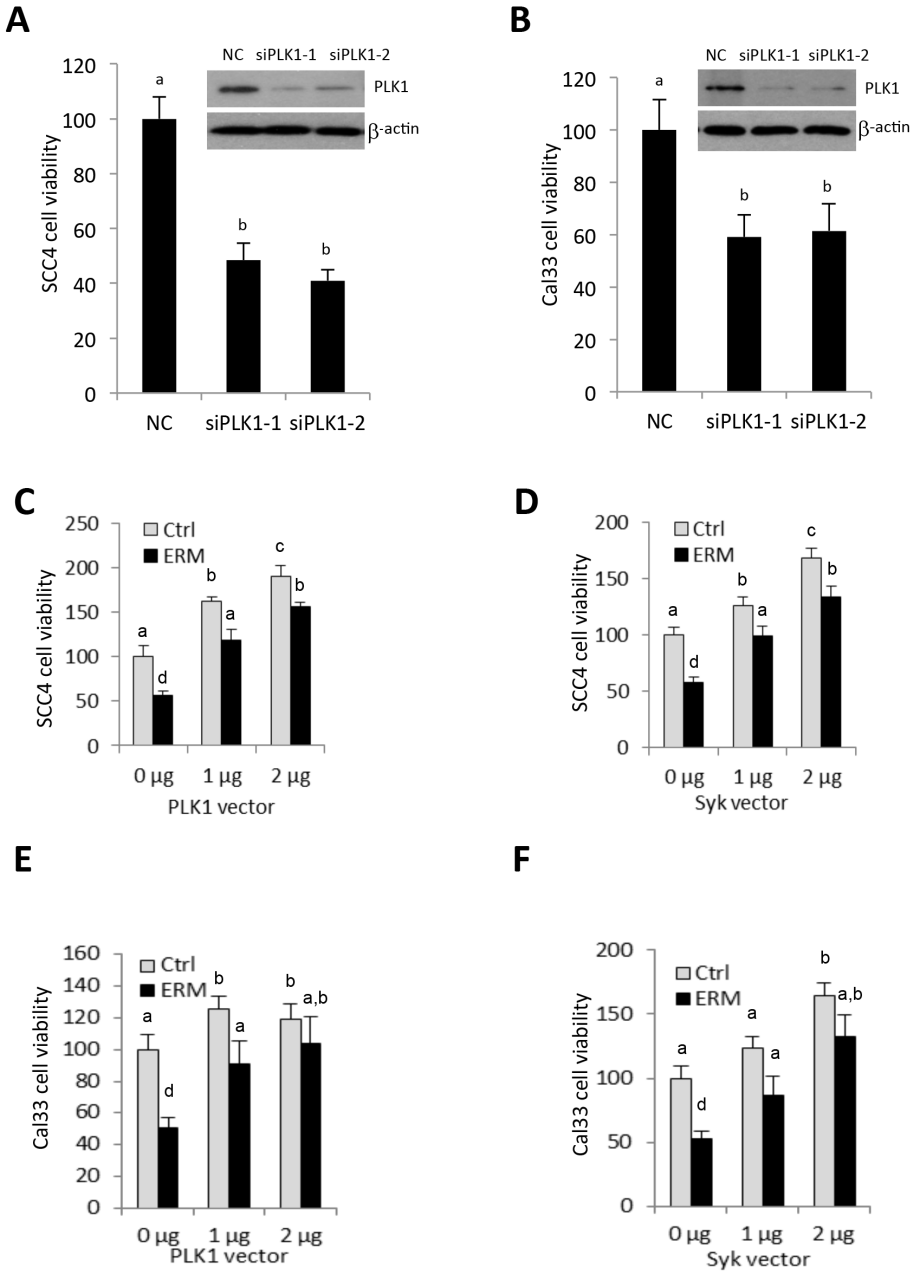
PLK1 siRNA mimics ER maleate effect on inhibition of cell viability, and PLK1 and Syk partially rescue ER maleate-reduced cell viability **A, B.** PLK1 siRNA transfection decreased cell viability in SCC4 (A) and Cal33 cells (B). Inset: siRNA mediated knockdown of PLK1 expression was shown in both cells by western blot (A, B). β-actin served as a loading control. **C–F.** Overexpression of PLK1 and Syk increased cell viability in a dose dependent manner in SCC4 (C, E) and Cal33 cells (D-F) and partially rescued ER maleate (1 μM) reduced cell viability both cells (C-F). The bar graph data were presented as mean ± SEM; groups denoted by different letters represent a significant difference at *p* < 0.05 (ANOVA followed by Fisher's LSD test).

Knockdown of PLK1 expression using two siRNA oligos individually targeting different regions of PLK1 mRNA showed a significant decrease in viability of SCC4 and Cal33 cells (Figure 8A, 8B), with western blot confirming PLK1 siRNA knockdown effect (Inset of Figure 8A, 8B), suggesting that inhibition of PLK1 correlated with the response to ER maleate treatment. Further. PLK1 overexpression induced cell viability in a dose dependent manner and partially or completely rescued ER maleate reduced cell viability in SCC4 and Cal33 cells (Figure 8C, 8E). Similar results were observed with Syk overexpression (Figure 8D, 8F).

### ER maleate is a chemosensitizer of platinum drugs

In comparison with ER maleate (0.5, 1.0 or 2.0 μM) or carboplatin (25μM or 50μM) treatment alone, a combination of ER maleate and carboplatin at their respective doses caused a further significant reduction of SCC4 cell viability after 48 h incubation (Figure 1E) and reduced colony formation was observed after 9 days (Figure 1G). Cell apoptosis and death detected by Annexin V and AAD-7 double staining were enhanced with ER maleate (2μM) treatment for 24 h, 48 h or 72 h in SCC4 cells (Figure 3A–3B) and Cal33 cells (Figure 3D–3E) in the presence of carboplatin (25μM). The expression of apoptotic markers including cleaved PARP, caspase9 and caspase3 also was further induced in SCC4 and Cal33 incubated with ER maleate (0.5, 1.0 or 2.0 μM) in combination with carboplatin (25μM) for 48 h (Figure 4A, 4B and Supplementary Figure S1A–S1L). Importantly, treatment with combination of carboplatin (25μM) and ER maleate (0.5, 1.0 or 2.0 μM) for 48 h led to further inhibition of the ER maleate-suppressed expression of PLK1, Syk, cyclin D1 and EGFR in SCC4 and Cal33 cells (Figure 7K, 7L, and Supplementary Figure S2A–S2H). ER maleate downregulated pAkt and total Akt levels were further reduced whereas activated ERK activity further induced by combination treatment with carboplatin for 24 h. Taken together ER maleate in combination with carboplatin further inhibited cell proliferation and induced cell death, suggesting its potential as chemosensitizer of platinum drugs in oral cancer cells.

### *In vivo* characterization of anticancer potential of ER maleate

**Figure 9:**
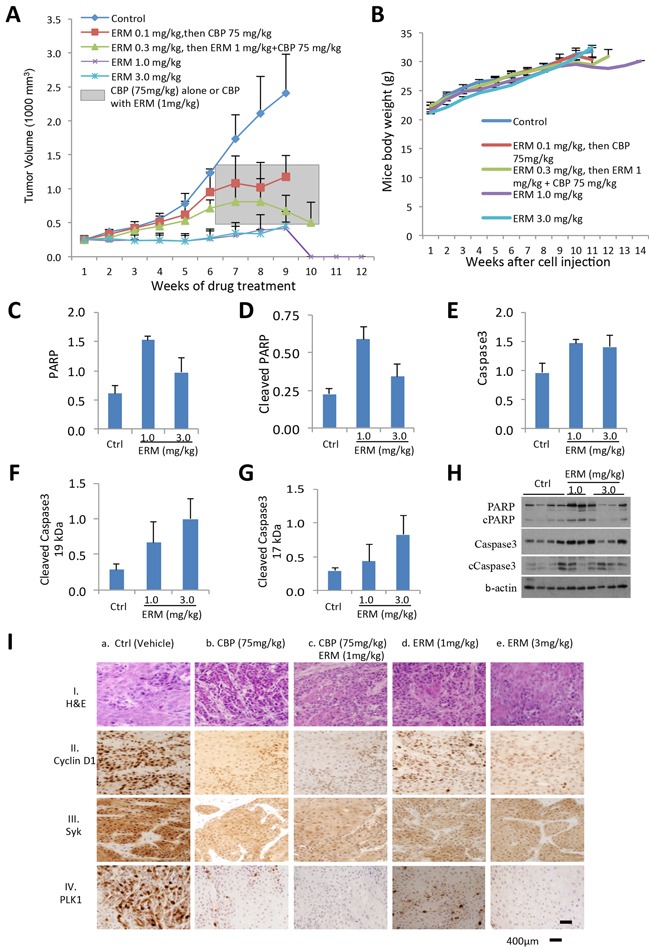
ER maleate anticancer potential in tumor xenograft mice model and IHC analysis of Syk and PLK1 in human patient OSCC **A.** ER maleate inhibits growth of tumor xenografts in mice. Cal33 cells were injected in the right flank of 6 weeks old immunocompromised mice (NOD/SCID/crl). ER maleate treatment was started after 3 weeks when tumor xenografts volume was about 250 mm^3^ with doses ranging from 0.1-3.0 mg/kg bwt for 10 weeks. Analysis of xenograft tumors from mice treated with ER maleate showed a dose dependent suppression of tumor growth within initial 6 weeks with an efficacious pharmacodynamic effect of complete inhibition of tumor growth at 1mg/kg bwt and 3mg/kg bwt by the 10^th^ week. From the 7^th^ week, the group of mice pre-treated with 0.1mg/kg bwt ER maleate was treated with CBP at 75mg/kg bwt and the group with ER maleate at 0.3mg/kg bwt within first 6 weeks received a combination treatment of ER maleate (1mg/kg bwt) and CBP (75mg/kg bwt) shown in the grey box. The combination treatment with ER maleate and CBP inhibited tumor growth *in vivo* from the 8^th^ week; in comparison, inhibition of tumor growth by CBP alone was lesser than in combination with ER maleate. **B.** Effect of ER maleate treatment on body weight of mice. Weekly measurements of mice body weight after Cal33 cell injection among different groups. **C–H.** Histogram of apoptotic markers in the frozen tumor tissues by western blot analysis showed the levels of PARP (C), cleaved PARP (D), caspase3 (E) and its two cleaved forms at 19 kDa (F) and 17 kDa (G) in xenograft tumor from groups receiving ER maleate of 1 mg/kg bwt or 3 mg/kg bwt compared to vechile control group. (H) Representative western blot showed the expression of PARP, cleaved PARP, caspase3 and its two cleaved forms at 19 kDa and 17 kDa. b-actin served as a loading control. **I.** Immunohistochemical analysis of Cyclin D1, Syk and PLK1 in tumor xenografts in immunocompromised mice. Panel I shows H&E stained tumor tissue sections in untreated control mice (a) and the treatment groups (b-e). Panels II, III and IV show nuclear Cyclin D1, Syk and PLK1 expression in untreated control mice (a); reduced Cyclin D1, Syk and PLK1 expression in CBP (75mg/kg bwt) treated tumors (b); combination of CBP (75mg/kg bwt) and ER maleate (1mg/kg bwt) shows further reduction in Cyclin D1, Syk and PLK1 (c); ER maleate treatment at 1mg/kg bwt and 3mg/kg bwt show reduced Cyclin D1, Syk and PLK1 expression in comparison with untreated controls (d and e), respectively. Original magnification is x400.

To characterize the anticancer potential of ER maleate in oral cancer and determine its tolerability and pharmacokinetic properties, an *in vivo* efficacy study was first performed by injecting Cal33 cells into immunocompromised mice (NOD/SCID/crl), and 3 weeks later followed by ER maleate treatment with doses ranging 0.1–3.0 mg/kg bwt (mice body weight) for 10 weeks. The tolerated dose for once-weekly administration of ER maleate was determined by the maximum dose that did not cause loss of body weight or other tolerability features (signs of illness, abnormal behavior including poor grooming, hunched posture, diarrhea, urine stains, and dehydration) in accordance with the Institutional humane endpoint guidelines. Doses within 0.1-3 mg/kg bwt range were well tolerated in oral tumor xenograft model. Analysis of xenograft tumors from mice treated with ER maleate (1mg/kg bwt and 3mg/kg bwt) showed an efficacious pharmacodynamic effect of complete inhibition of tumor growth by the 10^th^ week without significant body weight loss (Figure 9A, 9B). After 6 weeks of ER maleate treatment, mice groups treated with low dose of ER maleate (0.1mg/kg bwt and 0.3mg/kg bwt) showed similar increase in tumor volume as control group; the combined effects of ER maleate and carboplatin were then tested in these mice. From the 7^th^ week, the group of mice pre-treated with 0.1mg/kg bwt ER maleate was treated with carboplatin at 75mg/kg bwt, and the group receiving 0.3mg/kg bwt ER maleate for first 6 weeks received a combination of ER maleate (1mg/kg bwt) and carboplatin (75mg/kg bwt). Notably, ER maleate and carboplatin combination inhibited tumor growth *in vivo* from the 8^th^ week; carboplatin alone inhibited tumor growth marginally compared to control group (Figure 9A). ER maleate at 0.1-3mg/kg bwt, carboplatin at 75mg/kg bwt, or their combination, did not show any apparent toxicity to normal tissues by microscopic examination of hematoxylin-eosin (H&E) stained liver, kidney and heart tissues (Supplementary Figure S4), hematology and biochemistry analysis (Supplementary Table S3, S4). Analysis of the frozen tumor tissues from xenografts showed the levels of PARP, cleaved PARP, caspase3 and its two cleaved forms (19 kDa and 17 kDa) were increased in xenograft tumors from groups receiving ER maleate at a dose of 1 mg/kg bwt or 3 mg/kg bwt compared to the vehicle control group (Figure 9C–9H), suggesting that ER maleate induced cell apoptosis and inhibited cell proliferation *in vivo*. Importantly, our findings showed the chemosensitizing effect of ER maleate on carboplatin *in vivo* supporting the *in vitro* data and provided strong rationale for undertaking clinical studies to explore the efficacy of ER maleate in combination with carboplatin. IHC analysis of Cyclin D1, Syk and PLK1 expression in tumor xenografts and image analysis based quantitation of immunostained tissue sections using Visiopharm software showed 54%, 46% and 33% nuclear positivity in ER maleate treated group at 1mg/kg bwt, 21%, 31% and 15% in group at 3mg/kg bwt, 5%, 21% and 2.6% in group receiving combination of carboplatin and ER maleate, 41%, 35% and 19% in carboplatin treatment alone, and 82%, 92% and 77% in control group, respectively (Figure 9I). These *in vivo* studies provided evidence for downregulation of Cyclin D1, Syk and PLK1 in response to ER maleate treatment in oral tumor xenografts in support of PLK1 and Syk being novel molecular targets of ER maleate in OSCC.

### Overexpression of Syk and PLK1 in oral cancer patients

**Figure 10:**
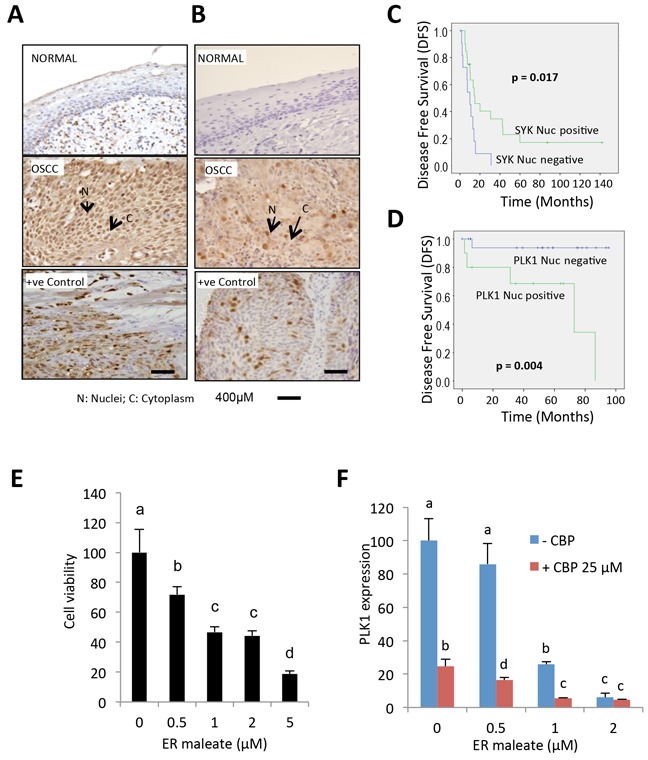
IHC analysis of Syk and PLK1 in tumor tissues and ER maleate effect on human OSCC derived cells **A.** IHC studies showed no detectable expression of Syk in normal oral mucosa. In OSCC, both nuclear (N) and cytoplasmic (C) expression of Syk were increased. Positive control OSCC tissue showing Syk overexpression was included in each batch of immunostaining. Original magnification is 400x. **B.** IHC analysis showed no detectable expression of PLK1 in normal oral mucosa. In OSCC, both nuclear (N) and cytoplasmic (C) expression of PLK1 were increased. Positive control OSCC tissue showing PLK1 overexpression was included in each batch of immunostaining. Original magnification is 400x. **C.** Kaplan-Meier survival analysis of Syk. OSCC patients showing nuclear Syk overexpression (Syk positive, Nuc_score_≥3) followed up over a period of up to 140 months showed a significant increase in mean disease free survival (DFS) (DFS = 41.03 months) as compared to patients who didn't show nuclear Syk positivity (Syk negative, Nuc_score_<3) (DFS = 10.58 months; *p* = 0.017). **D.** Kaplan-Meier survival analysis of PLK1. Survival analysis over a period of up to 100 months showed a significant reduction in mean DFS in OSCC patients overexpressing nuclear PLK1 (PLK1 positive, Nuc_score_≥3.5) (DFS =58.7 months) as compared to patients who didn't show nuclear PLK1 positivity (PLK1 negative, Nuc_score_<3.5) (DFS = 89.8 months; *p* = 0.004). **E.** Human OSCC derived cells showed a dose-dependent reduction in cell survival with ER maleate treatment for 48 h. **F.** PLK1 mRNA level was decreased by ER maleate in a dose dependent manner (0-2 μM) and this decreasing effect was further enhanced in OSCC derived cells with treatment of CBP at 25μM by qPCR assay. The bar graph data were presented as mean ± SEM; groups denoted by different letters represent a significant difference at *p* < 0.05 (ANOVA followed by Fisher's LSD test).

To assess the clinical significance of Syk and PLK1 in oral cancer, we compared nuclear (N) and cytoplasmic (C) expression levels of Syk and PLK1 proteins in normal oral mucosa and OSCCs. The clinical and IHC data of OSCC patients are shown in Supplementary Table S5 and S6. IHC studies revealed no detectable expression of Syk in normal oral mucosa (*n* = 16), while both nuclear (N) and cytoplasmic (C) Syk expression were observed in OSCC (*n* = 32) (Figure 10A). Kaplan-Meier survival analysis showed OSCC patients with nuclear Syk overexpression had a significant increase in mean disease free survival (DFS) (DFS = 41.03 months) as compared to patients with lower nuclear Syk expression (DFS=10.58 months, *p* = 0.017) (Figure 10C). The nuclear overexpression of PLK1 was observed in OSCC (*n* = 30) as compared to normal oral mucosa (*n* = 16) (Figure 10B). Significant reduction in mean DFS was observed in patients with nuclear PLK1 overexpression (DFS=58.7 months) as compared to patients with lower nuclear expression (DFS=89.8 months, *p* = 0.004) (Figure 10D). The independent effect of Syk and PLK1 also emerged as the most significant prognostic factors using multivariable Cox proportional hazard regression model, adjusted for age, T and N classification and clinical stage (Supplementary Table S7 and S8). Human OSCC derived cells showed dose-dependent reduction in cell survival with ER maleate treatment for 48 h (Figure 10E). PLK1 mRNA level was decreased by ER maleate (0-2 μM) and this decreasing effect was further enhanced in OSCC derived cells with treatment of Carboplatin at 25μM by qPCR (Figure 10F).

## DISCUSSION

We identified ER maleate as a potent anti-proliferative agent for OSCC. Our *in vitro* studies demonstrated novel effects of ER maleate treatment in oral cancer cells including G_2_/M arrest, blockade of cell division, aberrant mitosis and cells arrested as large polyploid cells. At molecular level, we observed downregulation of PLK1, Syk and CHEK2 in ER maleate treated cells that may account for deregulation of cell division. PLK1 is overexpressed during cancer development and plays a critical role in cell division as a major cell cycle regulator controlling entry into mitosis and regulating the spindle checkpoint [21, 22]. We observed that ER maleate inhibited PLK1 expression in SCC4 and Cal33 cells, suggesting PLK1 inhibition might mediate its anti-proliferative effect, cell cycle arrest in G_2_ phase, increase in polyploidy/aneuploidy with an increase in cell size. As a consequence, the mitotic cell cycle checkpoint may be affected and cell division blocked. Faulty chromosomal alignment and distribution may lead to cell tetraploidy/aneuploidy. Downregulation of PLK1 expression thus provides a potential mechanism for mitotic arrest and subsequent apoptosis in response to ER maleate in oral cancer cells. In support of mechanism of action of ER maleate, we have shown siRNA-mediated knockdown of PLK1 expression mimicked ER maleate mediated reduction in cell viability effects in OSCC. Further, these effects were partially rescued by overexpression of PLK1 in oral cancer cells. In support of our findings, shRNA-mediated decreased cellular PLK1 levels resulted in an increase of cells in G_2_/M phase, apoptosis and anti-proliferative effects [29]. Inhibition of PLK1 by siRNA in cancer cells *in vitro* has been shown to result in mitotic arrest and subsequent apoptosis demonstrating killing of cancer cells [30]. Interestingly, PLK1 effect is modulated by PI3K-Akt-mTOR [31] and MAPK pathways [32], both of which we have shown here were affected by ER maleate in oral cancer cells. Activation of MAPK signaling has been shown to decrease *PLK1* gene expression through p21/Waf1-mediated transcriptional repression targeted at a proximal promoter CDE/CHR site of *PLK1* [32]. Taken together, it is likely that inhibition of PLK1 actively contributes to ER maleate driven oral cancer cell death by modulating these pathways. *SYK* reported as an oncogene in B cell leukemia and lymphomas [33], head and neck cancer [18], ovarian cancer [34] and retinoblastoma [35], has made this kinase a popular target for the development of therapeutic agents. Exposure of cancer cells to EGF modulates the splicing pattern of *SYK* to promote the pro-survival isoform that is associated with cancer tissues *in vivo* [36] and EGFR activation also induces Syk phosphorylation [37]. Our data showed ER maleate inhibited EGFR expression and its phosphorylation, suggesting that inhibition of Syk expression might be partly mediated by the EGFR pathway. Further, our qPCR data revealed that ER maleate treatment reduced the levels of transcripts of the two kinases PLK1 and Syk, indicating downregulation at the transcriptional level or by modification of their mRNA stability as well.

Importantly, we showed that ER maleate downregulated CHEK2 in OSCC and this finding is important as CHEK2 is a critical regulator of G_2_/M cell cycle checkpoint [38]. Whether ER maleate-mediated decrease in CHEK2 expression leads to interruption of its interaction with CHEK1 and inhibits their roles in maintenance of G_2_/M checkpoint, cell mitosis and cell cycle progression in OSCC cells, thereby accounting for defective mitosis and polyploidy observed in OSCC cells, remains to be confirmed. Our findings provide more advanced knowledge on biochemical basis, potential targets, preclinical data and rationale for development of ER maleate for OSCC therapy.

Our studies showed that in addition to effects on Syk, PLK1 and CHEK2 expression, ER maleate also modulates PI3K/Akt/mTOR and MAPK signaling which are important pathways in OSCC. ER maleate induced cleavage of PARP, suggesting that induction of apoptosis was mediated by PARP pathway [39]. Activation of cleaved caspase3 suggested involvement of intrinsic mitochondrial pathway of apoptosis in ER maleate treated OSCC cells [40]. ER maleate induced pro-apoptotic BAD expression at mRNA level, further supporting the apoptotic effect of ER maleate. To gain insight into signaling of ER maleate induced cell apoptosis and death in OSCC cells, we investigated its effects on PI3K/Akt/mTOR pathway. We and others reported that aberrations of PI3K/Akt/mTOR pathway have been linked to various types of human cancer, including OSCC [41–45]. Activated PI3K/Akt signaling also controls cell growth and proliferation via mTOR associated protein S6 [45]. Our results revealed a decrease in pAkt (s473 and s308) levels supporting our data on ER maleate induced apoptosis in OSCC cells. Akt pathway promotes cell survival and proliferation by inhibiting the pro-apoptotic activity of Bad (pBad-S112) and hence activation of caspase9. ER maleate modulation of mTOR phosphorylation and its downstream molecule S6 phosphorylation (pS6) in SCC4 and Cal33 cells suggests PI3K/Akt/mTOR pathway plays an important role in mediating ER maleate action.

Extracellular MMPs play pivotal roles in oral cancer progression by promoting motility and invasion/metastasis of OSCC. Increased mRNA expression of MMP-9 is correlated with invasive behavior in UMSCC1 OSCC cells [46]. Several studies have demonstrated correlation between elevated TIMP1 levels and diminished MMP9 activity and invasiveness and migration [47]. In the present study, we did not observe significant change in MMP9 and TIMP1 in ER maleate treated SCC4 cells. However, ER maleate decreased MMP-1, -10, -12 and -13, with a parallel increase in TIMP2 expression on ER maleate treatment, suggesting TIMP2 may play a role in modulating ER maleate mediated regulation of MMPs. In support of our findings, upregulation of MMP-1, -10, -12 and -13 has also been shown in oral tongue SCC HSC-2 and Ca9-22 cells by genome-wide transcriptomic profiling [48]. Several studies have highlighted the dual functions of TIMP2 in regulating MMPs processing and inhibition of the active enzyme [49], and taken together with our findings we propose that TIMP2-MMPs contribute to ER maleate inhibited invasion and migration in SCC4 cells.

*In vivo* mouse xenograft studies revealed markedly tumor growth suppression in response to ER maleate treatment without noticeable reduction in weight or apparent toxicity in treated mice. IHC analysis of ER maleate treated tumor tissues showed a decrease in PLK1, Syk and Cyclin D1 expression, demonstrating that our *in vitro* findings were reproduced *in vivo* as well. These preclinical findings suggest that ER maleate has therapeutic effect on oral cancer *in vivo*.

Chemoresistance is one of the main causes for treatment failure in advanced cancers, where local therapies are insufficient. Carboplatin disrupts microtubule dynamics and is known to invoke the mitotic checkpoint. Our combinatorial studies using ER maleate and carboplatin provide the first evidence for their combination effects in SCC4 and Cal33 cells *in vitro* as well as in suppressing tumor growth in mice model *in vivo*. Carboplatin in combination with ER maleate induced apoptosis as evidenced by cleavage of PARP, caspases 3 and 9. ER maleate inhibition of PLK1 and CHEK2 has also been demonstrated to modulate the mitotic checkpoint, hence a cooperative effect with carboplatin could be expected. Thus our *in vitro* and *in vivo* studies underscore the chemosensitization potential of ER maleate for platinum drugs in OSCC.

Predictive biomarkers of sensitivity to targeted therapy are needed to identify the patient population most likely to respond to these agents. In an attempt to identify biomarkers that predict response to ER maleate, we showed ER maleate-mediated downregulation of Syk and PLK1 expression *in vitro* correlated with its tumor regression effects and decrease in Syk and PLK1 expression in treated mice xenografts *in vivo.* Our clinical studies using OSCC patients' tumor sections showed Syk and PLK1 overexpression in tumors that had prognostic relevance in follow up studies, suggesting their biomarker potential. The *in vitro* cytotoxicity assays using cells isolated from surgically resected OSCC patients' tumors showed ER maleate treatment decreased cell viability that was paralleled by reduction in PLK 1 mRNA levels. Taken together, these findings suggest PLK1/Syk have the potential to serve as intermediary biomarkers that predict response to ER maleate. However, this needs evaluation in a large number of OSCC patient samples treated with ER maleate to confirm their utility as intermediary biomarkers in future clinical trials.

In conclusion, our study provides *in vitro* and *in vivo* evidence for ER maleate as a promising potential anticancer agent for OSCC and chemosensitizer for platinum drugs. ER maleate downregulation of Syk and PLK1 may account for aberrant mitosis, cell arrested as large polyploid cells, blockade of cell division and cell death. Syk and PLK1 overexpression in OSCC patient and their association with poor prognosis as well as response to ER maleate suggest future clinical studies are warranted to explore an effective intervention of patients with Syk and PLK1 overexpressing OSCC by ER maleate, a potentially beneficial drug for personalized treatment.

## MATERIALS AND METHODS

### Cell culture and transient transfection

Human OSCC cell line, SCC4 was purchased from the American Type Culture Collection (Manassas, VA), MDA1986 was a kind gift from MD Anderson Cancer Centre (Houston, TX) in September, 2011, and HSC2 (JCRB0622) was obtained from Health Science Research Resources Bank, Japan in August, 2011. These cell lines were authenticated by DDC medical (Fairfield, OH) in November, 2011. The small molecule inhibitor libraries screening was performed in December, 2011 [15]. Cal33 was established from a moderately differentiated tongue SCC [50]. SCC4 and HSC2 are non-metastatic oral cancer cells of Caucasian and Asian origin, whereas MDA1986 and Cal33 cells have metastatic potential [42]. A new batch of SCC4 and Cal33 cell lines were obtained in 2013, their authentication was confirmed by DDC medical in March and September, 2014, respectively. All cells were used within 10 passages. Human OSCC derived epithelial cells were isolated from the surgical patient tumor tissue following the instruction of CELLection−Epithelial Enrich Dynabeads (Life Technologies, CA). Cells were grown in monolayer cultures in Dulbecco's modified Eagles medium (Life Technologies) supplemented with 10% fetal bovine serum (Sigma-Aldrich, St. Louis, MO) as described earlier [51]. Cell transfections were performed using Lipofectamin 2000 (Life Technologies). PLK1 plasmid was from OriGene (Rockville, MD) and Syk from Addgene (Cambridge, MA). PLK1 siRNA oligos shown in Supplementary Table S9 were synthesized by Genepharm (Shanghai, China).

### Cell cycle flow cytometry and imagestream analysis

Cell cycle analysis was performed using Gallios Flow Cytometer (Beckman Coulter, CA) and cell cycle modeling was carried out with ModFit LT software (Verity Software House, ME) [51]. Based on DNA content, image analysis features were used to distinguish mitotic events from G_2_ cells based on nuclear morphology. Amnis' imaging flow cytometry was run on ImageStreamX Mark II High resolution microscope and analyzed with IDEAS® Software (Amnis, Seattle, WA).

### Cell viability assay and annexin V assay

Cell viability was determined by MTT assay (Sigma-Aldrich). Apoptosis effect of ER maleate was evaluated by Annexin V and AAD-7 double staining as described earlier [51] using Gallios Flow Cytometer (Beckman Coulter).

### Colony formation and spheroid formation assay

For anchorage dependent clonogenicity assay, SCC4 (5,000 cells/well) were seeded in 6-well plates for ER maleate treatment (0.5 - 1 μM), with or without Carboplatin (25μM) for 48 h. Thereafter cells were incubated for up to 9 days in culture medium and colonies were counted. Hanging-drop culture was used for spheroid formation [52]. Briefly, SCC4 cells were incubated with ER maleate (0-5 μM) for 48 h, then 20 droplets, each containing 10,000 cells/15 μl, were plated on the inner surface of 10 cm petri dish cover. The covers were then inverted and placed on a dish containing 15 ml PBS. After 7 days of culture, spheroids were photographed and their size was measured using the ImageJ software.

### Cell invasion and migration assay

Cell invasion assay was performed in 24-well transwell plates (Costar™ Transwell™, Corning INC, NY) with 8μM polyethylene terephthalate membrane filters separating the lower and upper culture chambers. Cell migration was performed using wound healing assay [53].

### mRNA profiling and real-time quantitative PCR

Total RNA was extracted with Trizol (Invitrogen), 200ng of total RNA from each sample was used for Illumina mRNA profiling at Princess Margaret Genomics Centre (Toronto, ON). For real-time qPCR analysis, total RNA was reverse-transcribed into cDNA using oligo dT and Superscriptase III (Life Technologies) and real-time qPCR was performed in triplicate using SYBR green PCR on a 7900HT real-time PCR system (Applied Biosystems, CA) [54]. The qPCR primers are listed in Supplementary Table S9. Data were analyzed using SDS2.2 software (Applied Biosystems). Relative mRNA levels normalized by GAPDH were expressed using 2(−ΔΔCt) method.

### Western blot assay

Oral cancer cells (SCC4 and Cal33) were treated with ER maleate (0.5 – 2 μM). After 24 - 48 h, protein lysates were prepared in RIPA lysis buffer [54]. Equal amounts of proteins were subjected to SDS-PAGE and transferred to PVDF membranes. The membranes were incubated with primary antibody at 4°C overnight followed by incubation with HRP-conjugated secondary antibody (Supplementary Table S10). Immunoreactivity was detected using ECL (Pierce, Rockford, IL).

### *In vivo* mouse xenograft model

Mouse xenograft model was developed by subcutaneously injecting 6 × 10^6^ Cal33 cells in PBS in six weeks' old immunocompromised NOD/SCID/Crl mice in the right flank for tumor development as approved by Animal Ethics Committee of Mount Sinai Hospital (MSH) in accordance with Toronto Centre of Phenogenomics (TCP) guidelines. Mice with palpable tumors (250 mm^3^) were randomly assigned into 5 groups (4 mice/group), Group 1 (PBS control), Group 2 (ER maleate 0.1mg/kg bwt); Group 3 (ER maleate 0.3mg/kg bwt); Group 4 (ER maleate 1.0mg/kg bwt) and Group 5 (ER maleate 3mg/kg bwt). The mice were injected with ER maleate intraperitoneally (i.p.) weekly for 6 weeks. During this period, tumor volume and body weight were monitored weekly. Tumor volumes were measured using vernier callipers and calculated by (shortest diameter)^2^ × (longest diameter) × 0.5. At the end of experiment (or earlier if tumors exceeded 20% body mass), mice were sacrificed. Blood was collected from saphenous vein of mice for isolating serum and toxicology studies before euthanization. Tumors and organs were harvested and stored in 10% buffered formalin for histological assessment using H&E staining or IHC analysis.

### Patients

This retrospective study was approved by Research Ethics Board (REB) of MSH, Toronto, Canada, and written consent was obtained from each patient. The Reporting Recommendations for Tumor Marker prognostic Studies (REMARK) criteria were followed [55]. Inclusion criteria: Patients with histopathological evidence of OSCC confirmed by a pathologist and known clinical outcome. Exclusion criteria: Patients diagnosed with cancer of the oral cavity but with no available follow-up data. Patient demographic, clinical, and pathological data were recorded as described earlier [56]. These included clinical TNM staging (Union International Center le Cancer TNM classification of malignant tumors 1998), histopathological grade, age and gender (Supplementary Tables S5, S6). Following these criteria, archived formalin fixed paraffin embedded (FFPE) tissue blocks of OSCC patients undergoing curative surgery were inducted into this study (*n* = 32, median age: 60.25 years; range: 30.33-80.27 years for Syk and n = 30, median age: 59 years; range: 39-85 years for PLK1; Supplementary Tables S5, S6). All OSCC patients were treated as per the National Comprehensive Cancer Network (NCCN) guidelines for head and neck cancers.

### Follow-up study

All OSCC patients were followed in the cancer follow-up clinics regularly for up to 142 months (mean 30 months, median 15.5 months) and time to recurrence was recorded in MSH. If a patient died, the survival time was censored at the time of death. Disease-free survivors were defined as patients free from clinical and radiological evidence of local, regional, or distant relapse at the time of last follow-up. Follow-up period was defined as the interval from the time when patient underwent first surgery to recurrence of cancer (for uncensored observations) or no recurrence at last consultation (for censored observations) (Supplementary Tables S5, S6).

### Immunohistochemistry

Serial FFPE tissue sections (4μm thickness) of OSCCs and normal oral tissues, or tumor xenografts were deparaffinized in xylene, hydrated through graded alcohol series, followed by antigen retrieval, endogenous peroxidase activity and non-specific binding blocking [57]. The sections were incubated with anti-Syk (1:500 dilution) or anti-PLK1 antibodies (1:100) (Santa Cruz Biotechnology, Santa Cruz, CA) for 1 h at room temperature. Slides were incubated with biotinylated secondary antibody for 20 min, followed by VECTASTAIN Elite ABC reagent (Vector labs, Burlingame, CA) using diaminobenzidine as chromogen and counterstained with hematoxylin. The primary antibody was replaced by isotype-specific non-immune mouse/rabbit IgG in negative controls. Representative 5 stained areas were scored by 2 evaluators independently, who were blinded to clinical outcome as described earlier [57]. These sections were scored as: 0, < 10% cells; 1, 11-30% cells; 2, 31-50% cells; 3, 51-70% cells; and 4, > 70% cells showed immunoreactivity; intensity was scored as : 0, none; 1, mild; 2, moderate; and 3, intense. The slides were scanned using Nanozoomer 2.0 (Hamamatsu Photonics K. K., Hamamatsu, Japan) and image analysis was performed using Visiopharm Integrator System software Ver. 4.6.3.857 (Visiopharm, Hoersholm, Denmark).

### Statistical analysis

For statistical analysis, data presented as mean ±SEM were compared using the Two-tailed Student *t*-test or one-way ANOVA using GraphPad Prism 6.0 and clinical data were analyzed by SPSS 22. A p-value < 0.05 was considered statistically significant. Kaplan Meier survival and Multivariate Cox regression analysis were performed using SPSS 22 to determine the prognostic value of Syk and PLK1 as biomarkers for OSCC patients.

## SUPPLEMENTARY FIGURES AND TABLES



## Abbreviations

ERM: ER maleate
CBP: carboplatin
BAD: Bcl-2 associated death promoter
PARP: Poly (ADP-ribose) polymerase
PI3K: Phosphatidylinositol-3 kinase
mTOR: Mammalian target of rapamycin
pAkt: phosphor-Akt
EGFR: Epidermal growth factor receptor
STAT3: Signal transducer and activator of transcription 3
NFκB: Nuclear factor kappa B
Syk: Spleen tyrosine kinase
PLK1: Polo-like kinase 1
PLK4: Polo-like kinase 4
CHEK2: Cell cycle check point kinase 2
MMP: Matrix metalloproteinases
ERK: Extracellular signal-regulated kinases
Dip: Diploid
Anu: Anueploid
IHC: Immunohistochemistry

## References

[1] Ferlay J, Soerjomataram I, Dikshit R, Eser S, Mathers C, Rebelo M, Parkin DM, Forman D, Bray F (2015). Cancer incidence and mortality worldwide: sources, methods and major patterns in GLOBOCAN 2012. Int J Cancer.

[2] Scully C, Bagan JV (2008). Recent advances in Oral Oncology 2007: imaging, treatment and treatment outcomes. Oral Oncol.

[3] Argiris A, Karamouzis MV, Raben D, Ferris RL (2008). Head and neck cancer. Lancet.

[4] Nagao T, Chaturvedi P, Shaha A, Sankaranarayanan R (2011). Prevention and early detection of head and neck squamous cell cancers. J Oncol.

[5] Audrey RCB (2012). Head and Neck: Squamous cell carcinoma: an overview. Atlas Genet Cytogenet Oncol Haematol.

[6] Gomez DR, Zhung JE, Gomez J, Chan K, Wu AJ, Wolden SL, Pfister DG, Shaha A, Shah JP, Kraus DH, Wong RJ, Lee NY (2009). Intensity-modulated radiotherapy in postoperative treatment of oral cavity cancers. Int J Radiat Oncol Biol Phys.

[7] Budach W, Hehr T, Budach V, Belka C, Dietz K (2006). A meta-analysis of hyperfractionated and accelerated radiotherapy and combined chemotherapy and radiotherapy regimens in unresected locally advanced squamous cell carcinoma of the head and neck. BMC Cancer.

[8] Gibson MK, Li Y, Murphy B, Hussain MH, DeConti RC, Ensley J, Forastiere AA (2005). Randomized phase III evaluation of cisplatin plus fluorouracil versus cisplatin plus paclitaxel in advanced head and neck cancer (E1395): an intergroup trial of the Eastern Cooperative Oncology Group. J Clin Oncol.

[9] Pignon JP, le Maitre A, Bourhis J (2007). Meta-Analyses of Chemotherapy in Head and Neck Cancer (MACH-NC): an update. Int J Radiat Oncol Biol Phys.

[10] Brana I, Siu LL (2012). Locally advanced head and neck squamous cell cancer: treatment choice based on risk factors and optimizing drug prescription. Ann Oncol.

[11] Harari PM, Wheeler DL, Grandis JR (2009). Molecular target approaches in head and neck cancer: epidermal growth factor receptor and beyond. Semin Radiat Oncol.

[12] Le Tourneau C, Siu LL (2008). Molecular-targeted therapies in the treatment of squamous cell carcinomas of the head and neck. Curr Opin Oncol.

[13] Matta A, Ralhan R (2009). Overview of current and future biologically based targeted therapies in head and neck squamous cell carcinoma. Head Neck Oncol.

[14] Martens-de Kemp SR, Dalm SU, Wijnolts FM, Brink A, Honeywell RJ, Peters GJ, Braakhuis BJ, Brakenhoff RH (2013). DNA-bound platinum is the major determinant of cisplatin sensitivity in head and neck squamous carcinoma cells. PLoS One.

[15] Srivastava G, Matta A, Fu G, Somasundaram RT, Datti A, Walfish PG, Ralhan R (2015). Anticancer activity of pyrithione zinc in oral cancer cells identified in small molecule screens and xenograft model: Implications for oral cancer therapy. Mol Oncol.

[16] Geahlen RL (2014). Getting Syk: spleen tyrosine kinase as a therapeutic target. Trends Pharmacol Sci.

[17] Ghotra VP, He S, van der Horst G, Nijhoff S, de Bont H, Lekkerkerker A, Janssen R, Jenster G, van Leenders GJ, Hoogland AM, Verhoef EI, Baranski Z, Xiong J, van de Water B, van der Pluijm G, Snaar-Jagalska E (2015). SYK is a candidate kinase target for the treatment of advanced prostate cancer. Cancer Res.

[18] Luangdilok S, Box C, Patterson L, Court W, Harrington K, Pitkin L, Rhys-Evans P, P Oc, Eccles S (2007). Syk tyrosine kinase is linked to cell motility and progression in squamous cell carcinomas of the head and neck. Cancer Res.

[19] Archambault V, Glover DM (2009). Polo-like kinases: conservation and divergence in their functions and regulation. Nat Rev Mol Cell Biol.

[20] Macurek L, Lindqvist A, Lim D, Lampson MA, Klompmaker R, Freire R, Clouin C, Taylor SS, Yaffe MB, Medema RH (2008). Polo-like kinase-1 is activated by aurora A to promote checkpoint recovery. Nature.

[21] Degenhardt Y, Lampkin T (2010). Targeting Polo-like kinase in cancer therapy. Clin Cancer Res.

[22] Weiss L, Efferth T (2012). Polo-like kinase 1 as target for cancer therapy. Exp Hematol Oncol.

[23] Awada A, Dumez H, Aftimos PG, Costermans J, Bartholomeus S, Forceville K, Berghmans T, Meeus MA, Cescutti J, Munzert G, Pilz K, Liu D, Schoffski P (2015). Phase I trial of volasertib, a Polo-like kinase inhibitor, plus platinum agents in solid tumors: safety, pharmacokinetics and activity. Invest New Drugs.

[24] Lin CC, Su WC, Yen CJ, Hsu CH, Su WP, Yeh KH, Lu YS, Cheng AL, Huang DC, Fritsch H, Voss F, Taube T, Yang JC (2014). A phase I study of two dosing schedules of volasertib (BI 6727), an intravenous polo-like kinase inhibitor, in patients with advanced solid malignancies. Br J Cancer.

[25] Yim H (2013). Current clinical trials with polo-like kinase 1 inhibitors in solid tumors. Anticancer Drugs.

[26] Brandwein JM (2015). Targeting polo-like kinase 1 in acute myeloid leukemia. Ther Adv Hematol.

[27] Rudolph D, Impagnatiello MA, Blaukopf C, Sommer C, Gerlich DW, Roth M, Tontsch-Grunt U, Wernitznig A, Savarese F, Hofmann MH, Albrecht C, Geiselmann L, Reschke M, Garin-Chesa P, Zuber J, Moll J (2015). Efficacy and mechanism of action of volasertib, a potent and selective inhibitor of Polo-like kinases, in preclinical models of acute myeloid leukemia. J Pharmacol Exp Ther.

[28] Rudolph D, Steegmaier M, Hoffmann M, Grauert M, Baum A, Quant J, Haslinger C, Garin-Chesa P, Adolf GR (2009). BI 6727, a Polo-like kinase inhibitor with improved pharmacokinetic profile and broad antitumor activity. Clin Cancer Res.

[29] Spankuch B, Kurunci-Csacsko E, Kaufmann M, Strebhardt K (2007). Rational combinations of siRNAs targeting Plk1 with breast cancer drugs. Oncogene.

[30] Strebhardt K (2010). Multifaceted polo-like kinases: drug targets and antitargets for cancer therapy. Nat Rev Drug Discov.

[31] Zhang Z, Hou X, Shao C, Li J, Cheng JX, Kuang S, Ahmad N, Ratliff T, Liu X (2014). Plk1 inhibition enhances the efficacy of androgen signaling blockade in castration-resistant prostate cancer. Cancer Res.

[32] Zhang J, Wang S, Kern S, Cui X, Danner RL (2007). Nitric oxide down-regulates polo-like kinase 1 through a proximal promoter cell cycle gene homology region. J Biol Chem.

[33] Buchner M, Fuchs S, Prinz G, Pfeifer D, Bartholome K, Burger M, Chevalier N, Vallat L, Timmer J, Gribben JG, Jumaa H, Veelken H, Dierks C, Zirlik K (2009). Spleen tyrosine kinase is overexpressed and represents a potential therapeutic target in chronic lymphocytic leukemia. Cancer Res.

[34] Yu Y, Gaillard S, Phillip JM, Huang TC, Pinto SM, Tessarollo NG, Zhang Z, Pandey A, Wirtz D, Ayhan A, Davidson B, Wang TL, Shih Ie M (2015). Inhibition of Spleen Tyrosine Kinase Potentiates Paclitaxel-Induced Cytotoxicity in Ovarian Cancer Cells by Stabilizing Microtubules. Cancer Cell.

[35] Zhang J, Benavente CA, McEvoy J, Flores-Otero J, Ding L, Chen X, Ulyanov A, Wu G, Wilson M, Wang J, Brennan R, Rusch M, Manning AL, Ma J, Easton J, Shurtleff S (2012). A novel retinoblastoma therapy from genomic and epigenetic analyses. Nature.

[36] Prinos P, Garneau D, Lucier JF, Gendron D, Couture S, Boivin M, Brosseau JP, Lapointe E, Thibault P, Durand M, Tremblay K, Gervais-Bird J, Nwilati H, Klinck R, Chabot B, Perreault JP (2011). Alternative splicing of SYK regulates mitosis and cell survival. Nat Struct Mol Biol.

[37] Wu NL, Huang DY, Wang LF, Kannagi R, Fan YC, Lin WW (2016). Spleen Tyrosine Kinase Mediates EGFR Signaling to Regulate Keratinocyte Terminal Differentiation. J Invest Dermatol.

[38] Bruno T, De Nicola F, Iezzi S, Lecis D, D'Angelo C, Di Padova M, Corbi N, Dimiziani L, Zannini L, Jekimovs C, Scarsella M, Porrello A, Chersi A, Crescenzi M, Leonetti C, Khanna KK (2006). Che-1 phosphorylation by ATM/ATR and Chk2 kinases activates p53 transcription and the G2/M checkpoint. Cancer Cell.

[39] Gobeil S, Boucher CC, Nadeau D, Poirier GG (2001). Characterization of the necrotic cleavage of poly(ADP-ribose) polymerase (PARP-1): implication of lysosomal proteases. Cell Death Differ.

[40] McIlwain DR, Berger T, Mak TW (2013). Caspase functions in cell death and disease. Cold Spring Harb Perspect Biol.

[41] Molinolo AA, Marsh C, El Dinali M, Gangane N, Jennison K, Hewitt S, Patel V, Seiwert TY, Gutkind JS (2012). mTOR as a molecular target in HPV-associated oral and cervical squamous carcinomas. Clin Cancer Res.

[42] Ahn KS, Sethi G, Sung B, Goel A, Ralhan R, Aggarwal BB (2008). Guggulsterone, a farnesoid X receptor antagonist, inhibits constitutive and inducible STAT3 activation through induction of a protein tyrosine phosphatase SHP-1. Cancer Res.

[43] Hayes DN, Grandis JR (2015). Comprehensive genomic characterization of head and neck squamous cell carcinomas. Nature.

[44] Lui VW, Hedberg ML, Li H, Vangara BS, Pendleton K, Zeng Y, Lu Y, Zhang Q, Du Y, Gilbert BR, Freilino M, Sauerwein S, Peyser ND, Xiao D, Diergaarde B, Wang L (2013). Frequent mutation of the PI3K pathway in head and neck cancer defines predictive biomarkers. Cancer Discov.

[45] Molinolo AA, Amornphimoltham P, Squarize CH, Castilho RM, Patel V, Gutkind JS (2009). Dysregulated molecular networks in head and neck carcinogenesis. Oral Oncol.

[46] Sun L, Diamond ME, Ottaviano AJ, Joseph MJ, Ananthanarayan V, Munshi HG (2008). Transforming growth factor-beta 1 promotes matrix metalloproteinase-9-mediated oral cancer invasion through snail expression. Mol Cancer Res.

[47] Ramer R, Hinz B (2008). Inhibition of cancer cell invasion by cannabinoids via increased expression of tissue inhibitor of matrix metalloproteinases-1. J Natl Cancer Inst.

[48] Ye H, Yu T, Temam S, Ziober BL, Wang J, Schwartz JL, Mao L, Wong DT, Zhou X (2008). Transcriptomic dissection of tongue squamous cell carcinoma. BMC Genomics.

[49] Butler GS, Butler MJ, Atkinson SJ, Will H, Tamura T, Schade van Westrum S, Crabbe T, Clements J, d'Ortho MP, Murphy G (1998). The TIMP2 membrane type 1 metalloproteinase ‘receptor’ regulates the concentration and efficient activation of progelatinase A. A kinetic study. J Biol Chem.

[50] Gioanni J, Fischel JL, Lambert JC, Demard F, Mazeau C, Zanghellini E, Ettore F, Formento P, Chauvel P, Lalanne CM (1988). Two new human tumor cell lines derived from squamous cell carcinomas of the tongue: establishment, characterization and response to cytotoxic treatment. Eur J Cancer Clin Oncol.

[51] Matta A, DeSouza LV, Ralhan R, Siu KW (2010). Small interfering RNA targeting 14-3-3zeta increases efficacy of chemotherapeutic agents in head and neck cancer cells. Mol Cancer Ther.

[52] Ye G, Fu G, Cui S, Zhao S, Bernaudo S, Bai Y, Ding Y, Zhang Y, Yang BB, Peng C (2011). MicroRNA 376c enhances ovarian cancer cell survival by targeting activin receptor-like kinase 7: implications for chemoresistance. J Cell Sci.

[53] Fu G, Ye G, Nadeem L, Ji L, Manchanda T, Wang Y, Zhao Y, Qiao J, Wang YL, Lye S, Yang BB, Peng C (2013). MicroRNA-376c impairs transforming growth factor-beta and nodal signaling to promote trophoblast cell proliferation and invasion. Hypertension.

[54] Fu G, Peng C (2011). Nodal enhances the activity of FoxO3a and its synergistic interaction with Smads to regulate cyclin G2 transcription in ovarian cancer cells. Oncogene.

[55] McShane LM, Altman DG, Sauerbrei W, Taube SE, Gion M, Clark GM (2005). REporting recommendations for tumour MARKer prognostic studies (REMARK). Br J Cancer.

[56] Tripathi SC, Matta A, Kaur J, Grigull J, Chauhan SS, Thakar A, Shukla NK, Duggal R, Choudhary AR, Dattagupta S, Sharma MC, Ralhan R, Siu KW (2011). Overexpression of prothymosin alpha predicts poor disease outcome in head and neck cancer. PLoS One.

[57] Kaur J, Matta A, Kak I, Srivastava G, Assi J, Leong I, Witterick I, Colgan TJ, Macmillan C, Siu KW, Walfish PG, Ralhan R (2014). S100A7 overexpression is a predictive marker for high risk of malignant transformation in oral dysplasia. Int J Cancer.

