# Supplementary material for: ER maleate is a novel anticancer agent in oral cancer: implications for cancer therapy
Source: Oncotarget. 2016 Feb 26;7(13):17162–81. doi: 10.18632/oncotarget.7751 (PMC4941378; doi:10.18632/oncotarget.7751)
Supplement: Supplementary file 1 [file oncotarget-07-17162-s001.pdf]

SUPPLEMENTARY FIGURES AND TABLES

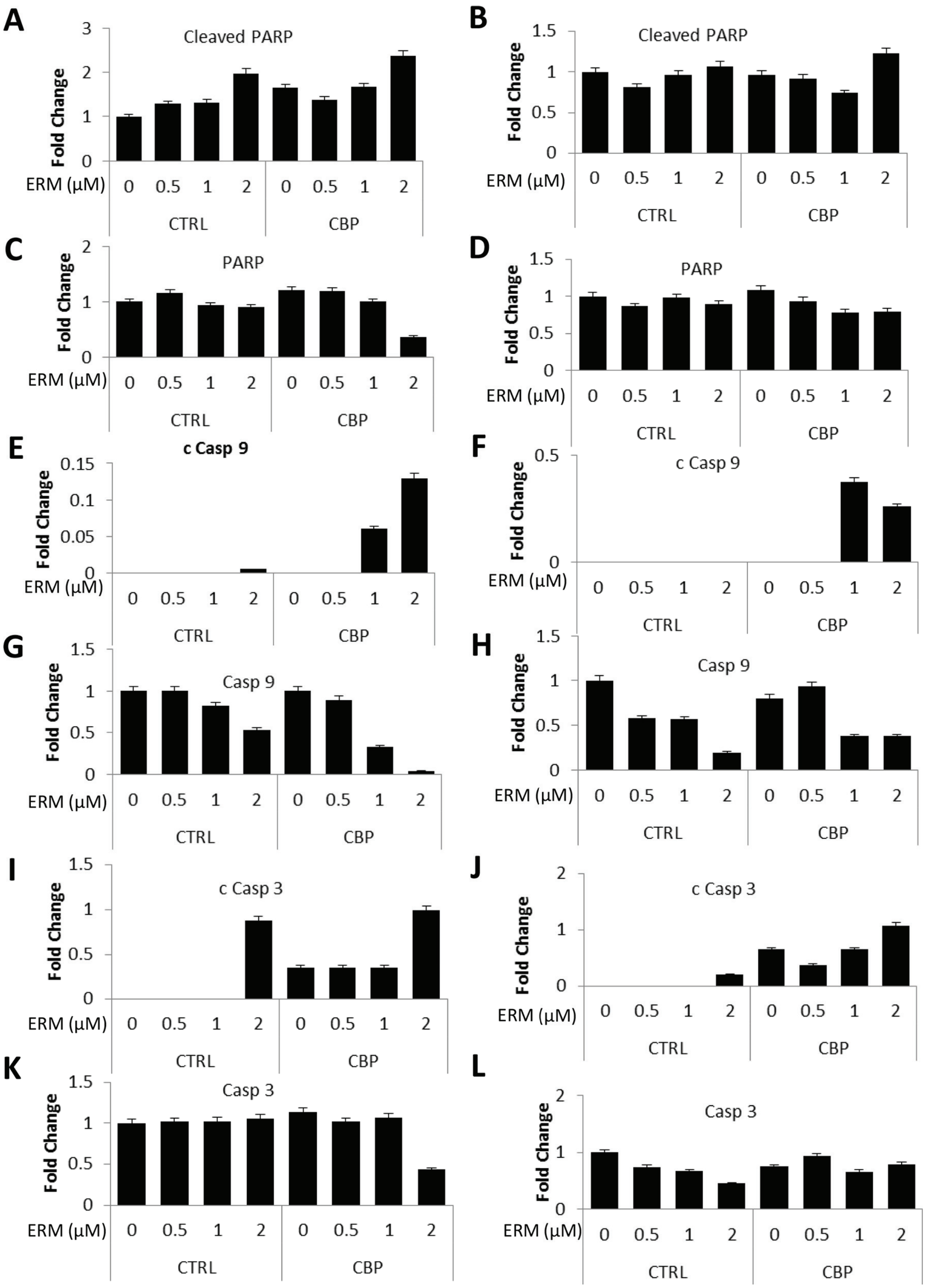

Supplementary Figure S1: Western blot densitometry analysis. Histograms of the western blot densitometry analysis of protein expression in SCC4 or Cal33 cells on treatment of ER maleate with or without presence of carboplatin (CBP 25μM). All the protein expression levels were normalised to GAPDHin comparison to untreated controls (NTC). **A.** Cleaved PARP in SCC4 cell; **B.** Cleaved Caspase 9 in Cal33 cell; **C.** PARP in SCC4 cells; **D.** PARP in Cal33 cell; **E.** Cleaved Caspase 9 in SCC4 cell; **F.** Caspase 9 in Cal33 cell; **G.** Caspase 9 in SCC4 cells; **H.** Caspase 9 in Cal33 cell; **I.** Cleaved Caspase 3 in SCC4 cell; **J.** Caspase 3 in Cal33 cell; **K.** Caspase 3 in SCC4 cells; **L.** Caspase 3 in Cal33 cell.

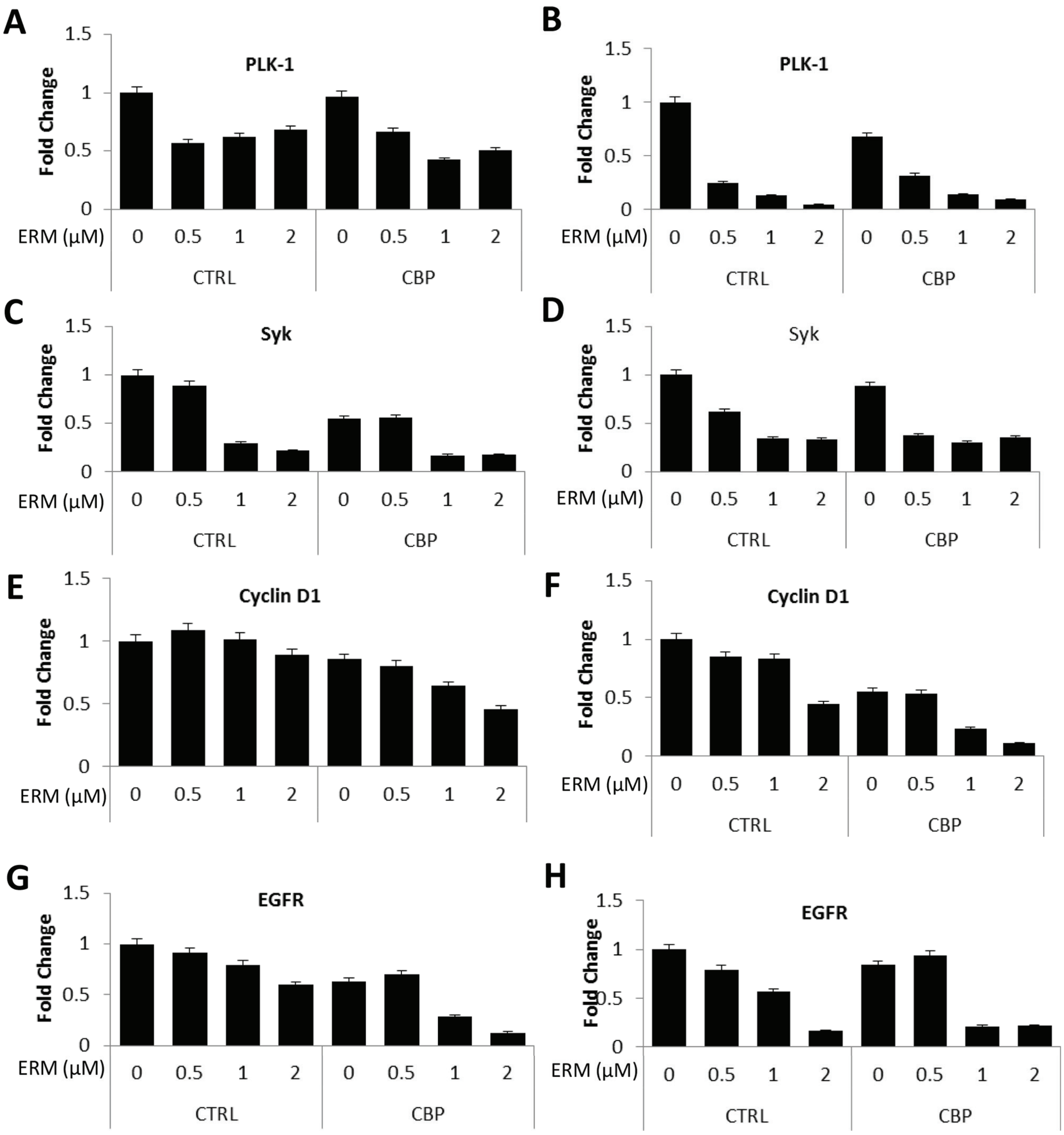

Supplementary Figure S2: Western blot densitometry analysis. Histograms of the western blot densitometry analysis of protein expression in SCC4 or Cal33 cells on treatment of ER maleate with or without presence of carboplatin (CBP 25µM). All the protein expression levels were normalised to GAPDH in comparison to untreated controls (NTC). **A.** PLK1 in SCC4 cell; **B.** PLK1 in Cal33 cell; **C.** Syk in SCC4 cells; **D.** Syk in Cal33 cell; **E.** Cyclin D1 in SCC4 cell; **F.** Cyclin D1in Cal33 cell; **G.** EGFR in SCC4 cells; **H.** EGFR in Cal33 cell.

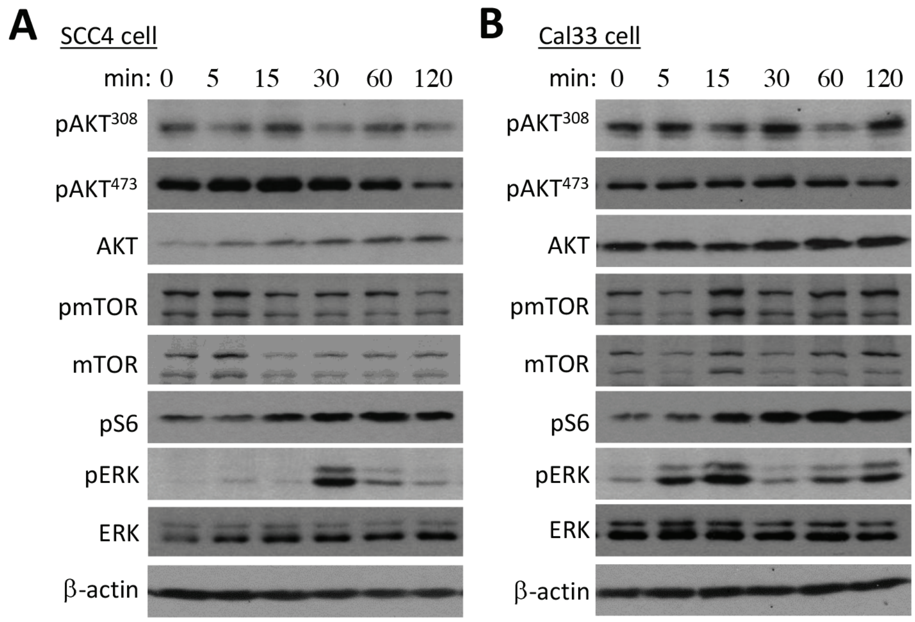

Supplementary Figure S3: ER maleate inhibits PI3K/Akt/mTOR and pERK signaling in OSCC cells. SCC4 and Cal33 cells were treated with ER maleate (2μM) for a short period (5-120 min). ER maleate treatment reduced the levels of $pAkt^{473}$ and $pAkt^{308}$ in SCC4 **A.** and Cal33 cells **B.** Similarly, phosphorylated mTOR (pmTOR) was also decreased whereas pS6 increased in both cells (A, B). On contrary, the activity of phosphorylated ERK (pERK) was induced by ER maleate in both cells (A, B). β-actin served as a loading control.

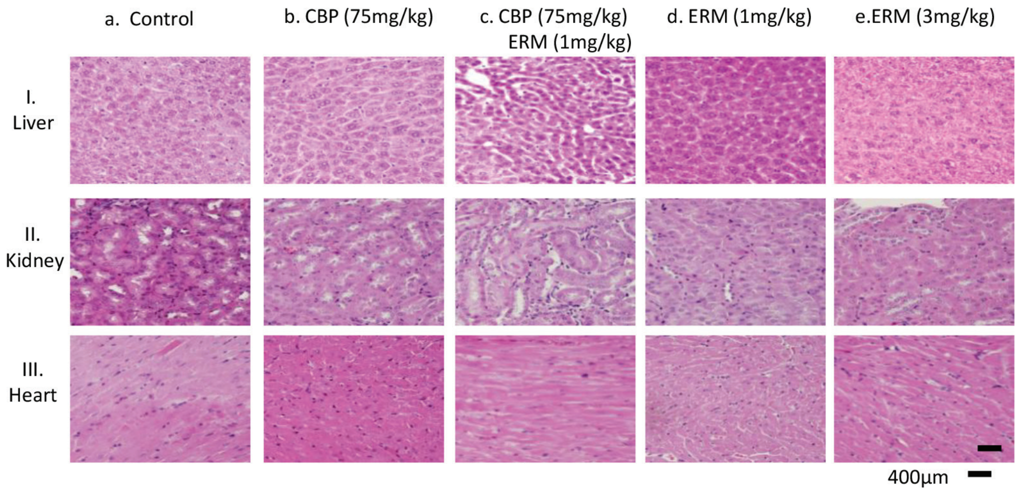

Supplementary Figure S4: ER maleate anticancer potential in tumor xenograft mice model. Hematoxylin and eosin stained liver, kidney and heart tissue sections. Histology of liver (I), kidney (II) and heart (III) tissues obtained at conclusion of the *in vivo* study. Hematoxylin-eosin (H&E) staining showed normal histology with no obvious signs of oncocytic necrosis or fibrosis observed in tissue sections (a-e) from different treatment groups. Original magnification is 400x.

Supplementary Table S1: ER maleate (ERM) effect on cell cycle in SCC4 cells using FACS and modfit analysis

SCC4\ERM	0 µM		0.5 µM		1 µM		2 µM	
Dip G_1		46.11%		43.25%		25.18%		16.56%
Dip G_2	95.42%	15.37%	93.88%	13.56%	82.84%	21.63%	70.82%	43.44%
Dip S		38.52%		43.19%		53.18%		40.00%
Anu G_1		37.98%		0		0		0
Anu G_2	4.58%	0	6.12%	0.54%	17.16%	0.05%	29.18%	0.32%
Anu S		62.05%		99.46%		99.95%		99.68%

Dip G_1: Diploid G_1 phase; Dip G_2: Diploid G_2 phase; Dip S: Diploid S phase; Anu G_1: Polyploid G_1 phase; Anu G_2: Polyploid G_2 phase; Anu S: Polyploid S phase

Supplementary Table S2: ER maleate (ERM) effect on cell cycle in Cal33 cells using FACS and modfit analysis

Cal 33\ERM	0 µM		0.5 µM		1 µM		2 µM	
Dip G_1		62.09%		56.17%		62.42%		59.23%
Dip G_2	100%	10.26%	100%	10.94%	94.43%	8.0%	43.86%	8.0%
Dip S		27.85%		32.89%		29.58%		32.77%
Anu G_1		n/a		n/a		43.55%		60.65%
Anu G_2	0	n/a	0	n/a	5.57%	5.15%	56.14%	6.08%
Anu S		n/a		n/a		51.3%		33.27%

Dip G_1: Diploid G_1 phase; Dip G_2: Diploid G_2 phase; Dip S: Diploid S phase; Anu G_1: Polyploid G_1 phase; Anu G_2: Polyploid G_2 phase; Anu S: Polyploid S phase

Supplementary Table S3: Clinical chemistry profiles among different mice groups with ER maleate or carboplatin treatment

	TEST	UNIT	DYNAMIC RANGE		NTC		ER 0.1mg/kg then CBP 75mg/kg		ER 0.3mg/kg then ER 1mg/kg and CBP 75mg/kg		ER 1mg/kg		ER 3mg/kg	
			Low	High	Mean	SD	Mean	SD	Mean	SD	Mean	SD	Mean	SD
ALB	Albumin	mg/dL	15	60	25.10	0.94	22.50	2.33	21.17	1.05	24.60	0.57	25.57	0.91
ALP	Alkaline Phosphatase	U/L	5	1500	26.95	4.62	24.13	2.90	15.57	2.42	24.15	2.05	30.43	1.81
ALT	Alanine Aminotransferase	U/L	3	500	23.70	4.30	24.13	6.45	31.37	24.04	127.75	96.94	21.73	2.15
AST	Aspartate Aminotransferase	U/L	3	1000	136.55	126.88	153.70	40.41	147.43	83.11	286.50	157.26	97.67	21.91
BUN	Urea Nitrogen	mg/dL	2	130	25.08	5.17	24.77	2.06	20.40	2.45	24.45	1.91	31.10	0.53
CREAT	Creatinine	mg/dL	0.2	25	0.18	0.03	0.16	0.04	0.16	0.03	0.20	0.04	0.15	0.03
B/C	BUN/Creatinine ratio**	-	0.08	650	135.70	12.68	158.98	26.16	127.96	30.38	126.04	18.08	205.68	37.87
TP	Protein, Total	mg/dL	30	120	44.50	2.29	43.23	2.29	44.10	3.22	43.85	1.91	44.53	2.06
CHOL	Cholesterol	mg/dL	NA	NA	118.05	13.03	119.50	8.87	128.57	19.38	122.70	8.63	117.40	7.99
HDL	High Density Lipoprotein	mg/dL	NA	NA	68.65	12.26	66.43	3.80	59.87	4.01	67.75	1.91	74.57	7.22
TRIG	Triglyceride	mg/dL	NA	NA	66.45	6.05	93.17	22.38	52.33	12.06	88.45	25.81	124.80	8.02
TBIL	Bilirubin, Total	mg/dL	0.1	30	0.31	0.14	0.21	0.04	0.16	0.03	0.23	0.01	0.22	0.02
GLU	Glucose	mg/dL	NA	NA	205.35	72.81	205.97	40.93	253.33	41.19	275.65	49.29	160.73	6.32
CA	Calcium, Total	mg/dL	4	18	9.81	0.61	9.93	0.40	10.88	1.18	9.34	0.75	11.63	1.24
PHOS	Phosphorus	mg/dL	1	20	8.41	2.14	6.56	2.73	6.35	1.42	7.27	2.31	6.02	1.22
NA	Sodium	mEq/L	50	200	150.13	5.86	148.70	4.42	146.80	10.78	130.30	3.68	144.83	10.83
K	Potassium	mEq/L	1	10	5.13	0.67	5.18	0.73	4.37	0.55	4.92	0.05	4.41	0.72
CL	Chloride	mEq/L	50	200	109.45	3.93	112.83	3.36	109.60	5.60	98.50	4.53	105.30	8.93
NA/K	Sodium/ Potassium ratio**	-	5	200	29.57	3.47	28.99	3.05	33.80	3.04	26.52	1.02	33.29	4.82

Supplementary Table S4: Hematology test among different mice groups with ER maleate or carboplatin treatment

Test	Reference		NTC		ER 0.1mg/kg then CBP 75mg/kg		ER 0.3mg/kg then ER 1mg/kg and CBP 75mg/kg		ER 1mg/kg		ER 3mg/kg	
			Mean	SD	Mean	SD	Mean	SD	Mean	SD	Mean	SD
RBC (10^12/L)	Red blood cells	7-13	7.16	0.54	4.91	1.13	6.47	1.00	6.12	1.09	7.25	0.21
Hgb (g/L)	Hemoglobin Concentration	13-16.9	103.75	7.63	77.33	17.95	103.00	8.72	89.00	12.73	108.00	4.76
HCT (L/L)	Hematocrit	0.474	0.37	0.02	0.27	0.06	0.38	0.03	0.32	0.08	0.38	0.01
MCV (fL)	Mean Corpuscular Volume	56	51.60	0.87	55.50	1.77	59.10	5.57	51.75	3.75	52.58	0.32
MCH (pg/cell)	Mean Corpuscular Hemoglobin	15.4	14.48	0.05	15.73	0.23	16.03	1.33	14.60	0.57	14.90	0.22
MCHC (g/L)	Mean Corpuscular Hemoglobin Concentration	275	280.75	3.59	283.67	13.61	272.00	4.36	282.50	30.41	283.50	3.70
PLT (10^9/L)	Platelet Count	350	478.50	440.99	185.67	60.17	404.67	59.37	190.50	26.16	603.25	166.48
WBC (10^9/L)	White Blood Cell	5-12	1.16	0.80	0.97	0.46	4.38	3.20	0.45	0.01	1.80	1.05
MPV (fL)	Mean Platelet Volume	351	5.70	1.00	6.40	0.46	5.67	0.46	6.95	0.35	4.93	0.28
RDW (%)	RBC Distribution Width	22	19.05	0.64	23.80	1.55	24.30	3.42	19.75	1.34	20.03	0.15
NE (10^9/L)	Neutrophil Count	NA	0.47	0.43	0.28	0.31	2.97	2.16	0.12	0.06	1.21	0.90
LY (10^9/L)	Lymphocyte Count	10.5	0.44	0.17	0.48	0.31	0.72	0.46	0.25	0.01	0.39	0.10
MO (10^9/L)	Monocyte Count	0.7	0.22	0.20	0.19	0.09	0.57	0.49	0.07	0.02	0.10	0.03
BA (10^9/L)	Basophil Count	NA	0.00	0.00	0.00	0.01	0.03	0.03	0.01	0.01	0.02	0.02
EO (10^9/L)	Eosinophil Count	0.2	0.03	0.02	0.02	0.01	0.09	0.09	0.02	0.01	0.07	0.05

Supplementary Table S5: Clinicopathological characteristics of OSCC patients and Syk expression

	N (%)
**HNSCC**	32
**Age (range, median)**	30.33 - 80.27(60.25)
**Sex**	
Male	22 (68.75)
Female	10 (31.25)
**Stage**	
I	12 (37.5%)
II	5 (15.63%)
III	2 (6.25%)
IV	12 (37.5%)
Unknown	1 (3.13%)
**Extra Capsular Invasion**	
Positive	6 (18.75%)
Negative	15 (46.88%)
Unknown	11 (34.38%)
**Perineural Involvement**	
Positive	6 (18.75%)
Negative	15 (46.88%)
Unknown	11 (34.38%)
**Vascular Involvement**	
Positive	3 (9.38%)
Negative	18 (56.25%)
Unknown	11 (34.38%)
**Follow-up Outcome**	
Positive	26 (81.25%)
Negative	6 (18.75%)
**Syk IHC Analysis**	
Nuclear positivity (≥ 3)	20 (62.5%)
Nuclear negativity (< 3)	12 (37.5%)

*No detectable Syk expression was observed in 16 normal oral tissues analyzed

Supplementary Table S6: Clinicopathological characteristics of OSCC patients and PLK1 expression

	N (%)
**HNSCC**	30
**Age (range, median)**	39-85 (59)
**Sex**	
Male	16 (53.33)
Female	14 (46.67)
**Stage**	
I	5 (16.67%)
II	5 (16.67%)
III	9 (30.0%)
IV	8 (26.67%)
Unknown	3 (10.0%)
**Extra Capsular Invasion**	
Positive	1 (3.33%)
Negative	29 (96.67%)
**Perineural Involvement**	
Positive	6 (20.0%)
Negative	24 (80.0%)
**Vascular Involvement**	
Positive	5 (16.67%)
Negative	25 (83.33%)
**Follow-up Outcome**	
Positive	6 (20.0%)
Negative	24 (80.0%)
**PLK1 IHC Analysis**	
Nuclear positivity (≥ 3.5)	10 (33.33%)
Nuclear negativity (< 3.5)	20 (66.67%)

*No detectable PLK1 expression was observed in 16 normal oral tissues analyzed

Supplementary Table S7: Multivariate Cox regression analysis for Syk expression in OSCC patients

OSCC	Kaplan-Meier survival analysis unadjusted p-value	Multivariate Cox regression analysis adjusted p-value	Hazard's ratio (H.R.)	95% C.I.
Syk_{Nuc}^{+}	**0.017**	**0.011**	**0.303**	**0.120 – 0.761**
Age	0.206	0.939	----	----
T Classification	0.385	0.374	----	----
Nodal Classification	0.068	0.165	----	----
Clinical Stage	0.135	0.109	----	----

Supplementary Table S8: Multivariate Cox regression analysis for PLK1 expression in OSCC patients

OSCC	Kaplan-Meier survival analysis unadjusted p-value	Multivariate Cox regression analysis adjusted p-value	Hazard's ratio (H.R.)	95% C.I.
$PLK1_{Nuc}^{+}$	**0.004**	**0.029**	**11.009**	**1.280-94.647**
Age	0.482	0.143	----	----
T Classification	0.714	0.223	----	----
Nodal Classification	0.514	0.056	----	----
Clinical Stage	0.319	0.514	----	----

Supplementary Table S9: List of Primers for real time PCR and siRNAs used in this study

No.	Primer	Sequence
1	PLK1 forward	5′-AGTCGACCACCTCACCTGTC-3′
	PLK1 reverse	5′-GCCCCTCACAGTCCTCAATA-3′
2	SYK forward	5′-GATGCTGGTTATGGAGATG-3′
	SYK reverse	5′-TCTATGATGTTCTTATCCTTGAC-3′
3	PLK4 forward	5′-AATCAAGCACTCTCCAATC-3′
	PLK4 reverse	5′-TGTGTCCTTCTGCAAATC-3′
4	GAPDH forward	5′-CAGAGCAAGAGAGGCATCCT-3′
	GAPDH forward	5′-TTGAAGGTCTCAAACATGAT-3′
5	siPLK1-1	5′-GCACAUACCGCCUGAGUCUtt-3′<br>3′-ttCGUGUAUGGCGGACUCAGA-5′
6	siPLK1-2	5′-CCACCAAGGUUUUCGAUUGtt-3′<br>3′-ttGGUGGUUCCAAAAGCUAAC-5′
7	siRNA negative control (NC)	5′-UUCUCCGAACGUGUCACGUtt-3′<br>3′-ttAAGAGGCUUGCACAGUGCA-5′

Supplementary Table S10: List of antibodies used in this study

S. No.	Antibody	Catalog No.	Company	Dilution
1	Syk	sc-1240	Santa Cruze	1:1000
2	PLK1	05-844	Millipore	1:1000
3	EGFR	ab93051	Abcam	1:500
4	PARP	9542	Cell Signaling	1:1000
5	Caspase 9	9502	Cell Signaling	1:1000
6	Cleaved Caspase 9	9505	Cell Signaling	1:500
7	Caspase 3	9962P	Cell Signaling	1:1000
8	Cleaved Caspase 3	9664P	Cell Signaling	1:500
9	Akt	C67E7	Cell Signaling	1:2000
10	p-Akt 308	C31E5E	Cell Signaling	1:500
11	p-Akt 473	D9E	Cell Signaling	1:1000
12	Bad	9292	Cell Signaling	1:500
13	pBad	S1127E11	Cell Signaling	1:1000
14	pmTOR	2974P	Cell Signaling	1:500
15	mTOR	2983P	Cell Signaling	1:1000
16	pS6	4857S	Cell Signaling	1:1000
17	Cyclin D1	ab134175	Abcam	1:1000
18	GAPDH	sc-365062	Santa Cruz	1:5000
19	β- actin	sc-47778	Santa Cruz	1:5000
